# Rainfall is associated with divorce in the socially monogamous Seychelles warbler

**DOI:** 10.1111/1365-2656.14216

**Published:** 2024-11-11

**Authors:** A. A. Bentlage, F. J. D. Speelman, J. Komdeur, T. Burke, D. S. Richardson, H. L. Dugdale

**Affiliations:** ^1^ Groningen Institute of Evolutionary Life Sciences University of Groningen Groningen The Netherlands; ^2^ School of Biological Sciences Macquarie University Sydney Australia; ^3^ School of Biosciences University of Sheffield Sheffield UK; ^4^ School of Biological Sciences University of East Anglia Norwich UK; ^5^ Nature Seychelles Mahé Republic of Seychelles

**Keywords:** climate window analysis, divorce, environmental conditions, habitat‐mediated hypothesis, passerine, rainfall, Seychelles warbler, social monogamy

## Abstract

1. Divorce—terminating a pair bond whilst both members are alive—is a mating strategy observed in many socially monogamous species often linked to poor reproductive success. As environmental factors directly affect individual condition and reproductive performance, they can indirectly influence divorce. Given current climate change, understanding how environmental fluctuations affect partnership stability has important implications, including for conservation. Yet, the relationship between the environment and divorce remains largely unstudied.
2. We examined the influence of temporal environmental variability on the prevalence of within‐ and between‐season divorce and the possible underlying mechanisms in a socially monogamous passerine.
3. Analysing 16 years of data from a longitudinal dataset, we investigated the relationship between rainfall and divorce in the Seychelles warbler (*Acrocephalus sechellensis*). First, we performed climate window analyses to identify the temporal windows of rainfall that best predict reproductive success and divorce. Then, we tested the effects of these temporal windows of rainfall on reproductive success and divorce and the influence of reproductive success on divorce whilst controlling for covariates.
4. Annual divorce rates varied from 1% to 16%. The probability of divorce was significantly associated with the quadratic effect of 7 months of total rainfall before and during the breeding season, with divorce increasing in years with low and high rainfall. This quadratic relationship was driven by a heavy rainfall event in 1997, as excluding 1997 from our analyses left a significant negative linear relationship between rainfall and divorce. Although the same temporal window of rainfall predicting divorce significantly influenced reproductive success, we found no significant correlation between reproductive success and divorce.
5. Our findings suggest that rainfall impacts divorce. Given that this effect is likely not directly mediated by reproductive success, we discuss other possible drivers. Although the 1997 super El Niño event shows how heavy rainfall may affect socially monogamous partnerships, more data are required to estimate the robustness of this effect. By adding to the growing body of literature showing that environmental conditions influence the stability of socially monogamous partnerships, we provide novel insights that may also be important for conservation efforts in times of climate change.

## Graphical abstract

To the best of our knowledge, our study is the first to show that extreme rainfall increases the prevalence of divorce in a socially monogamous population. Our findings add to the growing literature showing that harsh climates can affect partnership stability, which might be important for conservation efforts in times of climate change.

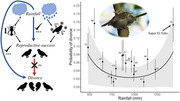

## 1 INTRODUCTION

Social monogamy, the mating system where individuals have one social breeding partner at a time, occurs in over 90% of birds (Lack, 1968). In these systems, maintaining a pair bond across multiple breeding seasons can improve reproductive success by reducing the costs associated with mate searching and enhancing mate familiarity (Choudhury & Black, 1994; Culina et al., 2020; Sánchez‐Macouzet et al., 2014). However, intra‐sexual competition often constrains mate selection, resulting in suboptimal partnerships. Suboptimal partnerships may be corrected through divorce, whereby a pair bond is terminated whilst both partners are alive (Choudhury, 1995), which can either increase, decrease, or have no effect on future reproductive success for one or both partners (Culina et al., 2015).

Divorce occurs in 92% of socially monogamous bird species (Jeschke & Kokko, 2008). With significant inter‐ and intra‐species variation in divorce rates (Black, 1996), several hypotheses have been proposed to explain what causes divorce (Choudhury, 1995). For instance, divorce may correct for genetic or behavioural incompatibilities within partnerships (Wilson et al., 2022) or enable individuals to choose a better‐quality partner, such as one with a higher dominance status or one that occupies a better territory than their previous partner (Blondel et al., 2000; Dhondt & Adriaensen, 1994; Otter & Ratcliffe, 1996). Here, one or both pair‐bonded individuals instigate divorce. Divorce can also be accidental, occurring due to temporal mismatches during migration (Gilsenan et al., 2017) or forced by the introduction of a third party (Jeschke et al., 2007). Related to several of these hypotheses, previous reproductive success and divorce are often correlated, with reproductive failure being a strong predictor of partnership termination (Culina et al., 2015). Notably, the effect of reproduction on divorce can vary depending on the stage of the breeding cycle, with failures at earlier breeding stages often being stronger predictors of divorce (Culina et al., 2015).

As climate patterns create suboptimal environmental conditions that affect individual condition and reproductive performance, they can influence divorce. The ‘habitat‐mediated’ hypothesis suggests divorce is more prevalent in unstable and lower‐quality environments (Blondel et al., 2000). This is because environmental factors can impact the decision‐making process underpinning divorce by misinforming individuals about their partnership's quality. For example, when partnerships perform poorly due to harsh environmental conditions, individuals within those partnerships may still attribute their poor performance to their chosen partner and not to the given circumstances (Ventura et al., 2021). Extreme weather can also increase physiological stress (Kitaysky et al., 2010), an important factor influencing mate selection (Husak & Moore, 2008). Given the rapid timing of climate change, marked by more frequent extreme weather events, such as droughts and floods, and increased global temperatures (NOAA National Centers for Environmental Information, 2022), which may limit possibilities for adaptation (Spooner et al., 2018), understanding how climate patterns affect the stability of socially monogamous partnerships is critical.

The relationship between the ecological environment and divorce remains largely unstudied, with only a handful of publications (Blondel et al., 2000; Botero & Rubenstein, 2012; Heg et al., 2003; Lerch et al., 2022; Ventura et al., 2021). Existing studies are primarily cross‐sectional, comparing the prevalence of divorce between species or populations of the same species. To the best of our knowledge, Ventura et al. (2021) is the only longitudinal study to have analysed the effects of climate‐driven environmental conditions on divorce within the same population, discovering that due to sea‐surface temperatures influencing food abundance and thus reproductive success, warmer sea‐surface temperatures increased the probability of divorce in black‐browed albatrosses (*Thalassarche melanophris*).

With climate change resulting in more frequent heavy rain and drought events (Marvel et al., 2019), we aimed to investigate the relationship between rainfall and divorce by analysing long‐term longitudinal data from the socially monogamous Seychelles warbler (*Acrocephalus sechellensis*), a passerine endemic to the Seychelles archipelago. Extreme rainfall negatively impacts the warblers' reproductive output (Borger et al., 2023; Komdeur, 1996a). As reproductive failures drive divorce in various bird species (Culina et al., 2015), including the Seychelles warbler (Speelman et al., 2024), we investigated whether (1) the temporal variability of rainfall affects the prevalence of divorce in the Seychelles warbler, (2) measures of reproductive success at four different stages of reproduction within the breeding season affect divorce, and (3) rainfall influences these four reproductive measures.

We predicted that extreme rainfall increases the prevalence of divorce (P1). As Seychelles warblers are insectivorous, low rainfall decreases food availability by impacting their prey's reproductive cycle (Komdeur, 1996a; Price, 1997). Conversely, high rainfall can affect the ability of birds to maintain optimal body temperatures and cause direct habitat and nest destruction (Kennedy, 1970; Wilson et al., 2004). Consequently, we predicted that low and high amounts of rainfall decrease reproductive success (P2). Specifically, due to decreased food availability, low rainfall impacts the ability of insectivorous birds to initiate breeding and produce a clutch (França et al., 2020). Then, due to decreased food availability and increased metabolic demands in heavy rainfall conditions, low and high rainfall impact nestling and fledgling survival (Heenan & Seymour, 2012; Monadjem & Bamford, 2009). The decreased reproductive success influenced by rainfall is predicted to increase the probability of divorce as reproductive success is used as a marker of a partnership's quality (P3). Overall, in line with the habitat‐mediated hypothesis, we predicted that divorce would be more prevalent following breeding seasons with poorer breeding conditions, with rainfall having a quadratic effect on divorce. Our findings may provide insights into how harsh environmental conditions affect reproduction and divorce in socially monogamous birds, which, in turn, can inform conservation efforts across multiple species in times of climate change, such as by informing population modelling.

## 2 MATERIALS AND METHODS

### 2.1 Study system

Since 1985, mark‐capture‐recapture data have been collected on the Seychelles warblers on Cousin Island (4°19′53.5′′ S 55°39′43.2′′ E). From 1997, >96% of the population has been caught and given unique identifiers using coloured bands and BTO‐numbered metal rings (Richardson et al., 2001). High annual resighting probabilities (98%) and no (<0.1%) inter‐island dispersal enable accurate individual‐level longitudinal measures of life‐history traits (Brouwer et al., 2006; Komdeur et al., 2004).

The insectivorous Seychelles warbler forms long‐term pair bonds and has a mean post‐fledgling lifespan of 5.5 years and a maximum observed lifespan of 19 years (Hammers & Brouwer, 2017; Raj Pant et al., 2020). Each of the ca. 110 territories on Cousin contains one dominant breeding pair. Dominant breeders are territorial, foraging most of their lives exclusively on their respective territories (Komdeur, 1991; Richardson et al., 2007). Cooperative breeding occurs: around half of the territories include 1–5 sexually mature subordinates, some of which (20% of males and 42% of females) act as helpers, providing alloparental care to the dominant breeders' offspring (Hammers et al., 2019; Richardson et al., 2003). Due to resource competition, helpers are more likely to be present in higher‐quality territories and can be maladaptive to breeders in lower‐quality territories (Komdeur, 1998).

The main Seychelles warbler breeding season spans from June to October, and the minor breeding season from December to March (Komdeur & Daan, 2005). Our analyses focused on the main breeding season as data on breeding statuses are limited in the minor breeding season, and although 30% of pairs breed (90% in main), breeding season type (main/minor) does not affect divorce in our study population (Speelman et al., 2024). Most (87%) clutches contain a single egg but can consist of up to three (Richardson et al., 2001). Co‐breeding subordinates often lay the additional eggs (Komdeur et al., 2004; Richardson et al., 2003). Insect abundance in a given month is predicted by rainfall 2 months prior (Komdeur, 1996a), likely cueing the onset of breeding to optimize food availability for nestlings. Although socially monogamous, there is a high rate of extra‐pair paternity (EPP), with 44% of offspring sired by males other than the social partner (Hadfield et al., 2006; Richardson et al., 2001). Lastly, parents often provide up to 3 months of post‐fledgling care to their offspring (Komdeur, 1991).

### 2.2 Data collection

We analysed data from 1997 to 2015 as social pairs have been monitored intensively since 1997, and rainfall measurements were only available until 2015. 1999 to 2001 were excluded due to limited fieldwork impacting the quality of partnership data required to classify divorces. During the main breeding season, all territories were monitored to determine the residency of ringed birds. Observations of foraging, singing, and non‐aggressive and aggressive social interactions were used to assign territory boundaries and group membership (Bebbington et al., 2017). The pair‐bonded male and female in a territory, determined based on their courtship and nesting behaviours, were defined as the dominant birds (Richardson et al., 2002). Breeding activity was monitored by following the dominant female for at least 15 mins every 1–2 weeks (Richardson et al., 2007). We identified the number of helpers, which influences reproductive success (Hammers et al., 2021), from nest watches of at least 60 min during the incubation and provisioning stages (van Boheemen et al., 2019). In case of a failed breeding attempt before an incubation or provisioning nest watch, subordinates could not be classified as helpers or non‐helpers, and the number of helpers was conservatively set to 0. The ages of unringed birds, usually caught before 1 year of age, were estimated using lay, hatch, or fledge dates and/or eye colour (Komdeur, 1991). DNA was extracted from caught individuals using brachial venipuncture blood samples (Richardson et al., 2001). Up to 30 microsatellite markers were genotyped to determine the relatedness between the dominant breeding pair and the parentage of offspring (see Supporting Information section ‘Pairwise relatedness’). Territory quality was measured using an index of insect availability, territory size, and foliage cover (see Supporting Information section ‘Territory quality’).

#### 2.2.1 Rainfall measurements

As rainfall data were not available from Cousin, we obtained mean monthly rainfall measurements from a weather station on Praslin (4°18′60.0″ S 55°43′59.9″ E), a neighbouring island ca. 1.5 km northeast of Cousin (Seychelles Meteorological Authority, 2016). Mean monthly and annual rainfall varied greatly during the study period (monthly range: 0.8–716 mm; annual: 1349–3410 mm).

### 2.3 Divorce classification

Partnerships were classified as divorced when there was a change in the identity of dominant breeders across breeding seasons whilst both previously pair‐bonded individuals were still alive. As breeding statuses were defined at the end of breeding seasons, divorce can occur within or between seasons. Temporary divorces, where pairs separate but reform after a breeding season, are rare (22 recorded cases: Speelman et al., 2024). As we were solely interested in comparing the years when partnerships did or did not divorce, we excluded the years when partnerships terminated due to partner deaths or translocations undertaken for conservation (Richardson et al., 2006; Wright et al., 2014). Our dataset included 416 males and 392 females in 1321 partnerships, 84 (6.4%) of which divorced.

### 2.4 Statistical analyses

All statistical analyses were performed in R 4.2.2 (R Core Team, 2022). Figures were created using *ggplot2* 3.4.1 (Wickham, 2016), and generalized linear mixed models (GLMMs) were run in *lme4* 1.1–31 (Bates et al., 2015). The over or underdispersion of models and residual spatio‐temporal autocorrelations were checked (none were found) using *DHARMa* 0.4.6 (Hartig, 2022). Collinearity was determined using *car* 3.1–1 (Fox & Weisberg, 2019), and all variance inflation factors (VIF) were <3.0. Model predictions for visualization were produced using *AICcmodavg* 2.3–1 (Mazerolle, 2020) and *ggeffects* 1.1–5 (Lüdecke, 2018). To facilitate model convergence, all explanatory variables were mean‐centred and divided by 1 standard deviation using the scale function in R. Unless stated otherwise, all estimates are given ± SE and the term ‘significant’ refers to statistical significance.

#### 2.4.1 Climate window analysis

Following Bailey and van de Pol (2016; see Supporting Information section ‘Climate window analysis’), we used *climwin* 1.2.3 to determine which temporal windows of rainfall best predicted divorce, reproductive success, and food availability. Previously, total rainfall from June to August was used to study the life‐history effects of rain in the Seychelles warbler (Borger et al., 2023). However, we analysed all possible temporal windows within 12 months before the end of the breeding season (28th of September), as we assumed that divorce is not an instantaneous decision but rather one that follows a long‐term decision‐making process influenced by multiple factors.

Firstly, we tested which temporal window of rainfall best predicted the probability of divorce (Y/N). Then, we tested which temporal windows of rainfall predicted measurements of reproductive success at four stages of reproduction: (1) The probability of attempting to breed—when a dominant breeding pair initiated nest building (Y/N); (2) The probability of producing a clutch—when the nest of a dominant breeding pair contained an egg (Y/N); (3) The probability of producing a fledgling—when an offspring fledged from the nest of a dominant breeding pair (Y/N); (4) The number of fledglings genetically related to the dominant female that survived until at least 3 months old (post‐fledgling care period)—classified as a continuous response variable (from now on named: ‘genetic fledglings’).

As all measurements of reproductive success could include offspring resulting from EPP, we assumed that male social partners were unaware of cuckolding and cared for offspring sired by other males as if they were their own. Although a minority (11% of offspring; Sparks et al., 2022), reproductive success measurements 1–3 could also include co‐breeders' offspring. Therefore, we included the number of genetic fledglings in our analyses to exclude offspring assigned to co‐breeding females.

Lastly, we tested which months of rainfall predicted territory quality (territory‐level measure) and insect abundance (population‐level measure; the mean number of insects found per unit leaf area across all monthly surveys). By investigating whether the temporal windows of rainfall best‐predicting divorce, reproductive success, and territory quality overlapped, we aimed to examine the links between rainfall, divorce, and possible drivers of divorce.

#### 2.4.2 Population‐level divorce rate

We used a quasi‐binomial generalized linear model (GLM) with a logit link function to model the annual population divorce rate as a function of rainfall and rainfall^2^. The measurement of rainfall included in the model was the total rainfall from the months that predicted divorce determined via the climate window analysis.

#### 2.4.3 Partnership‐level probability of divorce

Using a binomial GLMM with a logit link function, we modelled the probability of divorce as a function of rainfall, rainfall^2^, the number of offspring (genetic fledglings), the number of helpers, partnership length (in years), pairwise relatedness, male age (in years), male age^2^, female age, female age^2^, and population density (Hammers et al., 2019; Komdeur, 1994, 1996a, 1996b; Richardson et al., 2003; van Boheemen et al., 2019). All fixed effects were continuous variables. Next, we compared the effects of reproduction at four different stages—breeding attempted (Y/N), clutch produced (Y/N), fledgling produced (Y/N), and genetic fledglings produced (Y/N)—on divorce by including them in four separate models. We also tested our assumption that EPP did not affect divorce by including male and female EPP (Y/N)—when the dominant male or female was assigned parentage of offspring and the opposite‐sex parent assigned was not their social partner—in our model. Lastly, we included co‐breeder presence (Y/N) in our model to separate helper and co‐breeder effects on divorce.

#### 2.4.4 Partnership‐level probability of reproductive success

To explore the potential links between rainfall, reproductive success, and divorce, we examined whether rainfall during the months that best predicted divorce also influenced reproductive success. We used binomial GLMMs with logit link functions to model the probability of attempting to breed, producing a clutch, and producing a fledgling as functions of rainfall, rainfall^2^, the number of helpers, partnership length, pairwise relatedness, male age, male age^2^, female age, and female age^2^. Next, we used a Poisson GLMM with a log link function to model the effect of the same fixed effects on the number of genetic fledglings.

In all models, we tested whether partnership quality buffered the effects of rainfall by including interactions between partnership length and rainfall (all models) and reproductive success and rainfall (divorce models). We also included an interaction between rainfall and population density to test whether the increased availability of potential mates, resulting from extreme rainfall‐driven mortality, influenced divorce. Starting from a full model, we removed non‐significant quadratic terms and interactions in order of least significance to interpret first‐order effects. All models included the random effects: male ID, female ID, territory ID, and field period ID to control for birds sequentially performing worse or better than others and variable quality across territories and field periods (years).

## 3 RESULTS

### 3.1 Effect of rainfall on divorce

**FIGURE 1:**
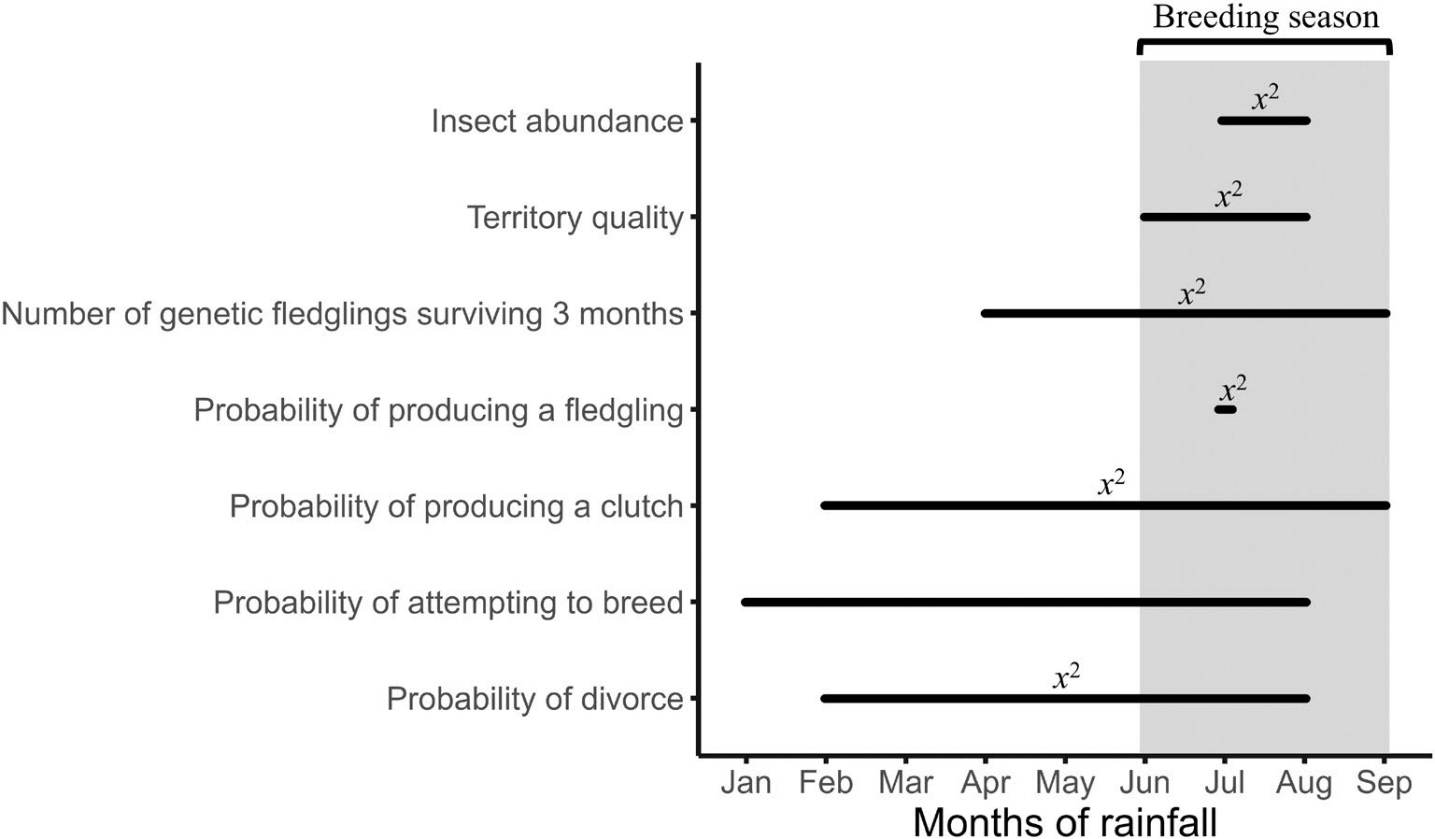
Temporal windows of rainfall that best predict seven response variables in the Seychelles warbler on Cousin Island (*n* = 1321 partnerships/15 years for insect abundance and territory quality [these data were not available in 2002]) as predicted by climate window analyses. Relationships between rainfall and the response variables were quadratic if indicated by *x* ^2^ and linear if not. The shaded area represents the main breeding season.

The mean Seychelles warbler annual divorce rate was 6.6 ± 1.1% and showed considerable inter‐annual variability (1.2%–15.6%; Figure S1a). Climate window analyses revealed that the quadratic effect of total rainfall from February to August best predicted the probability of divorce (Figure 1; Table S1).

**FIGURE 2:**
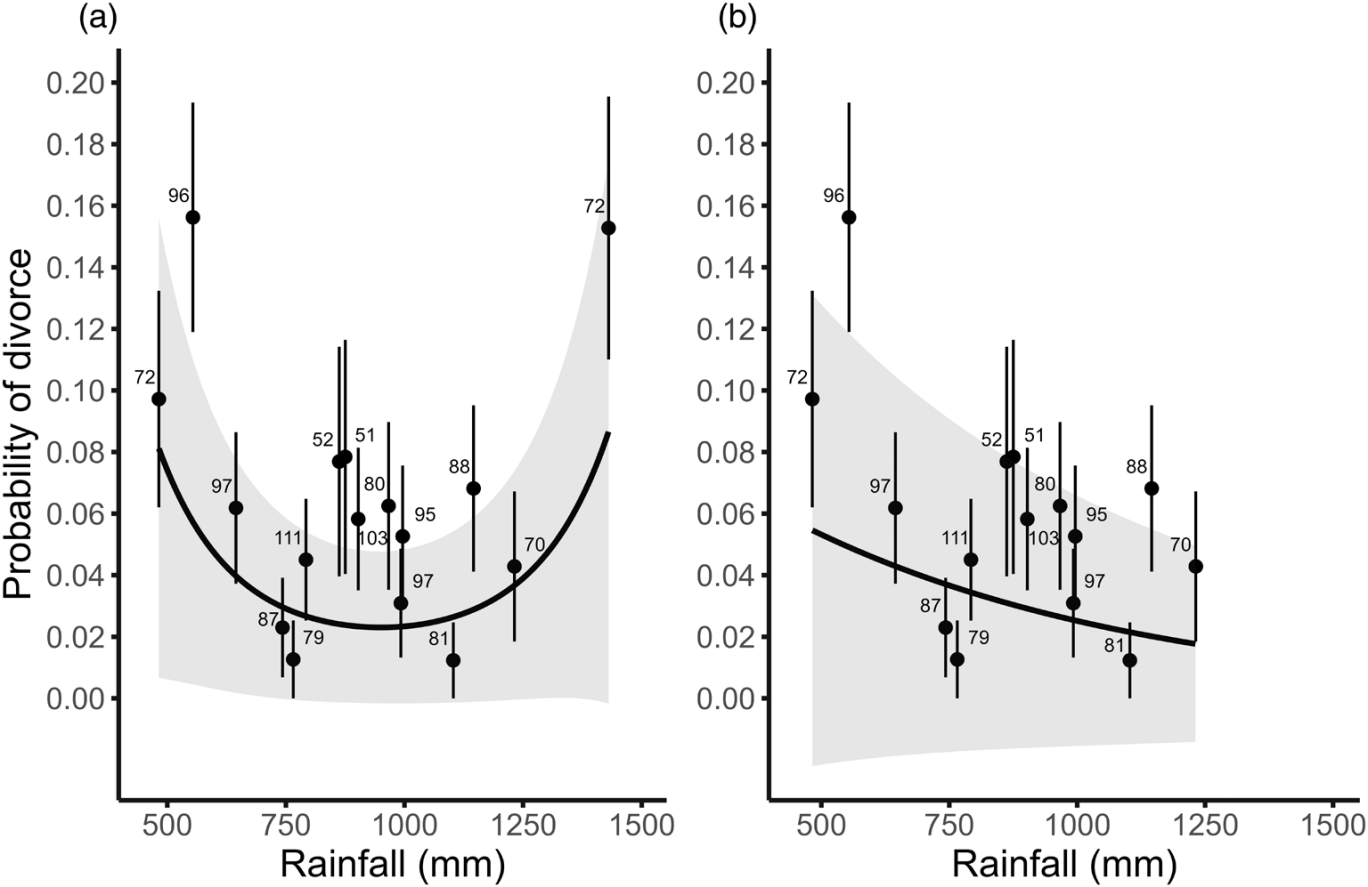
The effect of total rainfall from February to August on the probability of divorce in the Seychelles warbler on Cousin Island as predicted by binomial generalized linear mixed models. (a) includes the 1997 heavy rainfall event (*n* = 1321 partnerships) and (b) excludes 1997 (*n* = 1252 partnerships). The solid line represents the predicted probability of divorce and the shading indicates the 95% confidence intervals. Dots represent the mean observed divorce rate ± SE, and labels indicate the total number of partnerships observed in a given year.

At the population level, total rainfall from February to August had a significant quadratic effect on the annual divorce rate, which increased in years with low and high rainfall (GLM, estimate = 0.335 ± 0.091, *p*‐value = 0.003; Figure S1b). Rainfall effects explained 46.7% of the annual divorce rate's variance (*r* ^2^ = 0.467). At the partnership level, the quadratic effect of total rainfall from February to August significantly affected the probability of divorce (Table 1; Figure 2a). Notably, the quadratic relationship between rainfall and the probability of divorce was driven by extremely heavy rainfall in 1997, as excluding 1997 from the analysis revealed a significant negative linear relationship between rainfall and divorce (Tables S2 and S3; Figure 2b).

**TABLE 1:** Associations between the probability of divorce in the Seychelles warbler on Cousin Island with rainfall, the length of the partnership, the number of offspring, the relatedness of the breeding pair, the number of helpers, male age, female age, and population density.

Independent variables	Estimate	Standard error	95% Confidence interval	*p*‐Value
Intercept	−3.730	0.555	−4.817 to −2.642	**<0.001**
Rainfall	−0.152	0.113	−0.373 to 0.069	0.177
Rainfall^2^	0.336	0.090	0.160 to 0.512	**<0.001**
Partnership length	−0.455	0.231	−0.909 to −0.002	**0.049**
Number of offspring	−0.114	0.145	−0.399 to 0.171	0.431
Pairwise relatedness	0.162	0.141	−0.115 to 0.438	0.252
Number of helpers	−0.228	0.165	−0.552 to 0.095	0.166
Male age	0.076	0.170	−0.257 to 0.410	0.655
Female age	0.204	0.156	−0.102 to 0.511	0.192
Population density	0.018	0.133	−0.242 to 0.278	0.895

Random effects	Variance	Levels
Male ID	0.466	416
Female ID	0.600	392
Field period ID	0.000	16
Territory ID	0.282	158

*Note*: *n* = 1321 partnerships were analyzed using a binomial generalized linear mixed model. Significant *p‐*values are in bold.

### 3.2 Effects of reproductive success and other partnership qualities on divorce

Although we found a trend for reproductively successful partnerships to have lower divorce rates (Figure S2; Tables S4–S7), we found no significant correlations between the probability of divorce and measures of reproductive success (Figure S3; Tables S4–S7). However, the probability of divorce was significantly negatively correlated with partnership length (Table 1), with shorter partnerships having the highest probability of divorce. Notably, the correlation between rainfall and partnership length was non‐significant after excluding 1997 from the analyses (Tables S2 and S3). The mean partnership length in 1997 (0.8 ± 0.1) was considerably shorter than that of the full study period (2.2 ± 0.07). We also found a significant interaction between partnership length and rainfall (Table S8), with heavy rainfall increasing the probability of divorce in shorter but not longer‐lasting partnerships (Figure S4). However, this interaction was strongly influenced by outliers and subsequently removed from the final model (see Supporting Information section ‘Interaction between rainfall and partnership length’). EPP and co‐breeder presence were not associated with divorce (Table S10).

### 3.3 Effect of rainfall on reproductive success

#### 3.3.1 Breeding attempted

**FIGURE 3:**
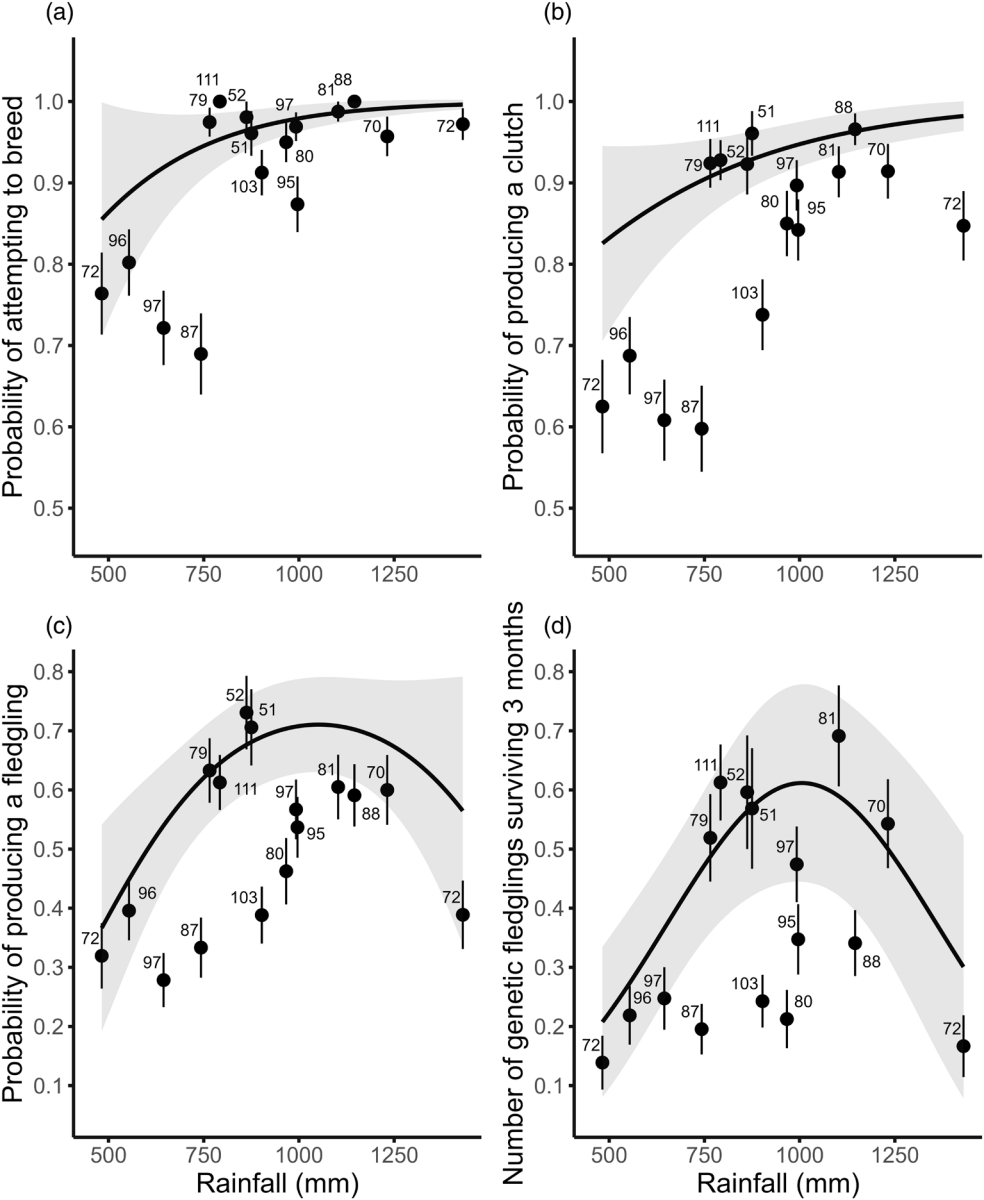
The effect of total rainfall from February to August on the probability of Seychelles warbler partnerships (*n* = 1321): (a) attempting to breed; (b) producing a clutch; (c) producing a fledgling; (d) the number of genetic fledglings surviving until 3 months old, as predicted by binomial (a–c) and Poisson (d) generalized linear mixed models. The solid line represents the predicted probability of divorce, and the shading indicates the 95% confidence interval. Dots represent the mean observed divorce rate ± SE, and labels indicate the total number of partnerships observed in a given year.

During the study period, 91% of partnerships attempted to breed. The probability of attempting to breed was best predicted by the linear increase in total rainfall from January to August (Figure 1; Table S1). The probability of attempting to breed was also significantly positively correlated with the months of rainfall best‐predicting divorce (February to August; Table S14; Figure 3a).

#### 3.3.2 Clutch produced

Overall, 83% of partnerships produced a clutch. The probability of producing a clutch was best predicted by the quadratic effect of rainfall from February to September (Figure 1; Table S1), decreasing in years with low and high rainfall. Although the climate window analysis predicted a quadratic relationship, we found a significant positive linear correlation between total rainfall from February to August and the probability of producing a clutch (Table S14; Figure 3b). The quadratic effect of rainfall was marginal (Table S15).

#### 3.3.3 Fledgling produced

Overall, 50% of partnerships produced a fledgling. The probability of producing a fledgling was best predicted by the quadratic effect of rainfall in July, the peak of egg‐laying (Figure 1; Table S1), where both low and high amounts of rain decreased fledgling success. We also found that total rainfall from February to August had a significant quadratic effect on the probability of producing a fledgling (Table S14). Again, intermediate levels of rainfall were associated with greater fledgling success (Figure 3c).

#### 3.3.4 Genetic fledglings

Approximately 32% of partnerships produced a genetic fledgling (mean number of genetic fledglings surviving = 0.40 ± 0.02). The number of genetic fledglings surviving was best predicted by the quadratic effect of rainfall from April to October (Figure 1; Table S1). Here, low and high amounts of rain decreased genetic offspring survival post‐fledgling care. Also, the number of genetic fledglings surviving was correlated with the quadratic effect of the total rainfall from February to August (Table S14). Again, greater genetic fledgling survival was associated with intermediate levels of rainfall (Figure 3d).

### 3.4 Effect of rainfall on food availability

A quadratic effect of total rainfall from June to August best predicted territory quality (Figure 1; Table S1). Although quadratic, this relationship was skewed to high rainfall correlating strongly with high territory quality, whilst low and intermediate rainfall were associated with lower territory quality (Figure S5a). An increase in insect abundance was best predicted by the increase in total rainfall from July to August (Figure 1; Figure S5b; Table S1).

## 4 DISCUSSION

### 4.1 Association between rainfall and divorce

As predicted (P1), rainfall had a quadratic effect on divorce in the Seychelles warbler, where low and high amounts of rain significantly increased the population‐level annual divorce rates and partnership‐level divorce probabilities. Extremely heavy rainfall in 1997 (a super ‘El Niño’ event) drove the association between high rainfall and divorce; excluding 1997 from the analyses left a negative relationship between rainfall and divorce. However, we consider 1997 to be biologically valid, as it shows the effects of the heavy rainfall events predicted to become more prevalent because of future climate change (Changnon, 2009; NOAA National Centers for Environmental Information, 2022; Pezza & Simmonds, 2008). Future investigations incorporating more extreme rainfall years would allow us to estimate the robustness of the quadratic effect. The main Seychelles warbler breeding season spans from June to October, and total rainfall from February to August best predicted divorce. Thus, if divorce is a decision informed by the costs and benefits of staying with a partner, it is likely reinforced by various drivers linked to rainfall between February and August.

Compared with the high divorce rates of some migratory birds, including the congeneric great reed warbler (*Acrocephalus arundinaceus*; 85%: Bensch & Hasselquist, 1991), the Seychelles warbler had a relatively low mean annual divorce rate (6.4%) similar to other birds with high site fidelity (3.7% in black‐browed albatrosses: Ventura et al., 2021), fitting with the prediction that birds with stable nesting sites are less likely to divorce (Choudhury, 1995). Nevertheless, inter‐annual divorce rates varied considerably and were significantly associated with rainfall. As rainfall is associated with food abundance (discussed below), our study is one of few to provide empirical evidence supporting the habitat‐mediated hypothesis of divorce (Ventura et al., 2021).

### 4.2 Association between rainfall and reproductive success

As predicted (P2), rainfall significantly influenced reproductive success. Borger et al. (2023) discovered that total rainfall from June to August had a quadratic effect on the number of genetic Seychelles warbler fledglings produced. We investigated this further by examining rain effects on all reproductive stages. Total rainfall from January to August and February to September best predicted the probability of attempting to breed, and producing a clutch, respectively. These reproductive measures were also significantly positively correlated with the temporal window of rainfall best‐predicting divorce. These large temporal windows support studies showing rain impacts birds' reproductive success by affecting the health of birds outside of the breeding season (Studds & Marra, 2007). Rainfall can impact individual condition and reproductive success by influencing food abundance (often insects), which explains why rainfall cues breeding for many birds (Cavalcanti et al., 2016; França et al., 2020; Lloyd, 1999).

On Cousin, the increase in total rainfall from June to August and July to August was associated with increased territory quality and population‐wide insect abundance, respectively. As most insects lay their eggs in water, drought significantly limits their development (Chen et al., 2019; Price, 1997), decreasing food availability for the warblers. As mean food availability at the end of the breeding season was best predicted by rainfall around the middle of the breeding season, our results support the 2‐month temporal window previously found by Komdeur (1996a). Thus, the Seychelles warbler likely uses rainfall to cue breeding to ensure adequate food availability for offspring. Consequently, by limiting the ability to invest in offspring, low rainfall decreases the probability of attempting to breed and producing a clutch.

The probability of producing a fledgling was predicted by rainfall in July, the month of peak egg‐laying, which is consistent with studies that found that the probability of fledging in birds correlated with rainfall during the hatchling period (Monadjem & Bamford, 2009; Schöll & Hille, 2020). Next, the number of genetic fledglings produced was best predicted by rainfall from April to September. Here, rainfall can directly affect fledgling survival or do so indirectly by impacting parental care during the months of post‐fledgling care. Total rainfall from February to August had a significant quadratic effect on both measures of fledgling success, where low and high amounts of rain decreased the probability of fledgling survival. Alongside the aforementioned effects of low rainfall on food availability, heavy rainfall, and the often accompanying strong winds, can be detrimental as they can destroy nests and make maintaining optimal body temperature difficult for birds (Kennedy, 1970; Wilson et al., 2004). As nestlings often lack fully developed feathers, hindering their ability to maintain body temperature, it can be detrimental to their survival if they get wet (Mertens, 1977; Newton, 1998). Similarly, heavy rainfall can increase the parental investment required to maintain optimal nest temperatures (Heenan & Seymour, 2012). If required parental investments increase during harsh weather conditions and their foraging ability is limited, they may face a trade‐off between provisioning and their health (Radford et al., 2001), impacting the survival of their offspring (Öberg et al., 2015).

### 4.3 Association between reproductive success and divorce

The temporal window of rainfall that predicted divorce overlapped with the temporal windows of rainfall predicting measures of reproductive success. All measurements of reproductive success were also significantly correlated with total rainfall from the months that best predicted divorce, and there was a trend for higher mean divorce rates in partnerships with lower reproductive success. Low reproductive success impacting divorce is in line with findings of previous studies (Culina et al., 2015; Mercier et al., 2021; Pelletier & Guillemette, 2022; Ventura et al., 2021), including in our study population (Speelman et al., 2024). However, when accounting for rainfall effects in our models, the direct effects of reproductive success on the probability of divorce were non‐significant. Thus, reproductive success may not influence divorce in the Seychelles warbler as predicted (P3), and rainfall may influence divorce through alternative pathways.

Physiological stress may influence divorce in the Seychelles warbler. Harsh environmental conditions and food scarcity can increase the concentration of stress hormones in birds (Kitaysky et al., 2010), which are positively associated with an individual's level of dissatisfaction with their social partner (Griffith et al., 2011). Although the role of stress in divorce is currently unknown for the Seychelles warbler, research shows that lower territory quality correlates with higher levels of oxidative stress because of increased foraging effort, especially during the early stages of reproduction (Komdeur, 1991, 1996b; van de Crommenacker et al., 2011). Thus, rainfall and its effects on food availability and parental investments could increase physiological stress in the Seychelles warbler. Individuals may associate their heightened physiological stress with their choice of partner, causing individuals in resource‐poor seasons to terminate partnerships regardless of reproductive output, signifying that stress could be the link between rainfall and divorce. Studies analysing relationships between stress markers, such as glucocorticoids (Sapolsky et al., 2000), rainfall (or other environmental effects), and divorce, are required to investigate this theory.

Divorce can be an adaptive strategy that improves reproductive success (Culina et al., 2015). In times of climate change, behavioural plasticity may help animals minimize the negative consequences of coping with rapid environmental changes (Beever et al., 2017). Our study introduces the possible consequences of climate change on partnership stability. However, further research into divorce consequences is required to determine whether rainfall‐driven divorce is adaptive and can help the species overcome climatic challenges. An understanding of whether rainfall influences divorce in good‐ or bad‐quality partnerships is currently lacking. If rainfall affects divorce by misinforming individuals about their partnership's quality, either through impacting stress or reproductive performance, divorce can occur in partnerships that may perform adequately in good conditions. Here, rainfall‐driven divorce can be maladaptive, making climate change a concern to the future of this species. In the Seychelles warbler, no short‐ or long‐term reproductive costs of divorce have been detected (Speelman et al., 2024). However, as this study did not test for divorce consequences in the context of environmental effects, studies disentangling divorce fitness consequences in poor and high‐quality years are required.

### 4.4 Non‐environmental associations with divorce

Older partnerships were less likely to divorce, fitting the prediction that divorce benefits are highest before individuals have gained the benefits associated with mate familiarity (Choudhury, 1995). Whilst behavioural incompatibilities between individuals can also manifest early in partnerships and influence divorce by impacting reproductive success (Wilson et al., 2022), we found no effect of partnership length on reproductive success. Consistent with studies showing that Seychelles warblers do not seem to avoid inbreeding (Eikenaar et al., 2008), we also found no effect of pairwise relatedness on divorce, indicating that inbreeding avoidance or other genetic incompatibilities (Hidalgo Aranzamendi et al., 2016) are unlikely drivers of divorce in our population. Notably, the effect of partnership length on divorce was non‐significant when excluding 1997 from the analysis. In 1997 population monitoring intensified, and much more of the Seychelles warbler population became identity‐tagged (>96% of the population; Richardson et al., 2001). The mean partnership length in 1997 was considerably shorter than in other years. This may be because the limited nature of the data prior to this year meant that partnership lengths were underestimated that year, and consequently, removing it led to the loss of the significant interaction. Other earlier years (1999–2001) were already excluded from the analyses due to reduced partnership‐level data—required to classify divorces—being collected in those years. Thus, biases in the data may drive the effect of partnership length on divorce.

## 5 CONCLUSIONS

We provide empirical evidence for an association between rainfall and divorce in a socially monogamous population, thereby contributing to a growing body of literature showing that harsh climates affect partnership stability. The prevalence of divorce in the Seychelles warbler was highest in years with low and high rainfall. We provide correlational evidence that this could result from rain impacting reproductive success, possibly by affecting food availability and parental trade‐offs between investing in current versus future reproductive success. We also discuss alternative explanations involving the role of physiological stress, an important avenue for further research in this and other species. Studies show that temperature influences divorce in birds, and now we find that rainfall does too. The climate can directly affect survival and indirectly influence population stability by restricting reproductive output. We do not yet understand whether rainfall‐driven divorce in the Seychelles warbler is adaptive, maladaptive, or neutral. Therefore, studying the consequences of divorce in this species may highlight to what extent plasticity in breeding behaviour can enable socially monogamous species to adapt to a rapidly changing world.

## AUTHOR CONTRIBUTIONS

Frigg J. D. Speelman and Hannah L. Dugdale conceived the study question. Agus A. Bentlage, Frigg J.D. Speelman, and Hannah L. Dugdale designed the hypotheses and methodology. Agus A. Bentlage and Frigg J. D. Speelman performed the data selection. Terry Burke, Jan Komdeur, David S. Richardson, and Hannah L. Dugdale maintain the long‐term dataset. David S. Richardson managed and undertook fieldwork over the period involved. Agus A. Bentlage analysed the data and wrote the manuscript with input from Frigg J. D. Speelman, Hannah L. Dugdale, David S. Richardson, and Jan Komdeur. All authors gave final approval for publication.

## FUNDING INFORMATION

Frigg Speelman was funded by a PhD scholarship from the University of Groningen and Macquarie University, the Lucie Burgers Foundation, and both the Ecology Fund Grant and Dobberke Grant from the Royal Netherlands Academy of Arts and Sciences. The long‐term data collection was supported by Natural Environment Research Council grants: NE/B504106/1, NE/F02083X/1, NE/I021748/1, NE/K005502/1, NE/P011284/1, and Dutch Research Council grants: 825.09.013, 823.01.014, 854.11.003, and 040.11.232.

## CONFLICT OF INTEREST STATEMENT

All authors have no competing interests.

## STATEMENT ON INCLUSION

The aid and input of local stakeholders from Nature Seychelles, who manage Cousin Island, in the conservation of, and research into, the Seychelles warbler is an important part of our work. As a result of this close interaction, underpinned by a memorandum of understanding, David Richardson is affiliated with Nature Seychelles, and we reciprocate by providing scientific support, intellectual input, and funding for facilities. Nature Seychelles is also included as an author affiliation in all Seychelles warbler publications.

## Supporting information


**Table S1.** Temporal windows of rainfall that best predict seven response variables in the Seychelles warbler on Cousin Island (*n* = 1321 partnerships/15 years for insect abundance and territory quality) as predicted by climate window analyses.
**Table S2.** Associations between the probability of divorce in the Seychelles warbler on Cousin Island with the total rainfall from February to August, the length of the partnership, the number of offspring, the relatedness of the breeding pair, the number of helpers, male age, female age, and population density. Data from 1997 were removed from this analysis and the non‐significant quadratic term of rainfall is included.
**Table S3.** Associations between the probability of divorce in the Seychelles warbler on Cousin Island with the total rainfall from February to August, the length of the partnership, the number of offspring, the relatedness of the breeding pair, the number of helpers, male age, female age, and population density. Data from 1997 were removed from this analysis and the non‐significant quadratic term is excluded.
**Table S4.** Associations between the probability of divorce in the Seychelles warbler on Cousin Island with rainfall, the length of the partnership, *breeding attempted*, the relatedness of the breeding pair, the number of helpers, male age, female age, and population density.
**Table S5.** Associations between the probability of divorce in the Seychelles warbler on Cousin Island with rainfall, the length of the partnership, *clutch produced*, the relatedness of the breeding pair, the number of helpers, male age, female age, and population density.
**Table S6.** Associations between the probability of divorce in the Seychelles warbler on Cousin Island with rainfall, the length of the partnership, *fledgling produced*, the relatedness of the breeding pair, the number of helpers, male age, female age, and population density.
**Table S7.** Associations between the probability of divorce in the Seychelles warbler on Cousin Island with rainfall, the length of the partnership, *genetic fledgling produced*, the relatedness of the breeding pair, the number of helpers, male age, female age, and population density.
**Table S8.** Associations between the probability of divorce in the Seychelles warbler on Cousin Island with the total rainfall from February to August, partnership length, the number of offspring, the relatedness of the breeding pair, the number of helpers, male age, female age, and population density.
**Table S9.** The number of available samples of Seychelles warbler partnerships that have been together for different lengths of time on Cousin Island.
**Table S10.** Associations between the probability of divorce in the Seychelles warbler on Cousin Island with rainfall, the length of the partnership, the relatedness of the breeding pair, the number of helpers, male age, female age, population density, male extra‐pair‐paternity (EPP; infidelity), female EPP, and co‐breeder presence (Y/N).
**Table S11.** Associations between the probability of divorce in the Seychelles warbler on Cousin Island with the total rainfall from February to August, partnership length, the number of offspring, pairwise relatedness, the number of helpers, male age, female age, and population density. Partnerships with a partnership length greater than 3 (scaled value) were removed from this analysis.
**Table S12.** Associations between the probability of divorce in the Seychelles warbler on Cousin Island with the total rainfall from February to August, partnership length, the number of offspring, pairwise relatedness, the number of helpers, male age, female age, and population density. Partnerships with a partnership length greater than 2 (scaled value) were removed from this analysis.
**Table S13.** Associations between the probability of divorce in the Seychelles warbler on Cousin Island with the total rainfall from February to August, breeding experience, the number of offspring, the relatedness of the breeding pair, the number of helpers, male age, female age, and population density.
**Table S14.** Associations between the probability of attempting to breed (model 1), producing a clutch (model 2), producing a fledgling (model 3), and the number of genetic fledglings surviving till at least three months old (model 4) in the Seychelles warbler on Cousin Island with rainfall, partnership length, pairwise relatedness, the number of helpers, male age, and female age.
**Table S15.** Associations between the probability of producing a clutch in the Seychelles warbler on Cousin Island with total rainfall from February to August, the length of the partnership, the relatedness of the breeding pair, the number of helpers, male age, and female age.
**Figure S1.** (a) Variability in the annual divorce rate of the Seychelles warbler on Cousin Island (*n* = 1321 partnerships) from 1997 to 2015. (b) The effect of rainfall on the annual divorce rate as predicted by a quasi‐binomial generalized linear model.
**Figure S2.** The probability of divorce for Seychelles warbler partnerships (*n* = 1321) that did or did not: (a) attempt to breed; (b) produce a clutch; (c) produce a fledgling; (d) produce a fledgling genetically related to the dominant female that survived till at the least three months old.
**Figure S3.** The coefficient estimates (dots) and 95% confidence intervals (bars) of four measures of reproductive success on the probability of divorce in the Seychelles warbler (*n =* 1321 partnerships) as predicted by binomial generalized linear mixed model.
**Figure S4.** The effect of total rainfall from February to August on the probability of divorce for Seychelles warbler partnerships (*n* = 1321) that have been together for different lengths of time on Cousin Island as predicted by a binomial generalized linear mixed model.
**Figure S5.** The effect of rainfall on: (a) territory quality (scaled 1:10,000); (b) insect abundance (the mean number of insects found per unit leaf area across all monthly surveys) on Cousin Island (*n* = 15 years), as predicted (solid line) by a *climwin* generated linear model.

## Data Availability

Data available from the University of Groningen dataverse https://doi.org/10.34894/GIAR3J (Bentlage et al., 2024).
