# Supplementary material for: Rainfall is associated with divorce in the socially monogamous Seychelles warbler
Source: J Anim Ecol. 2024 Nov 11;94(1):85–98. doi: 10.1111/1365-2656.14216 (PMC11730830; doi:10.1111/1365-2656.14216)
Supplement: Supplementary file 1 — Table S1. Temporal windows of rainfall that best predict seven response variables in the Seychelles warbler on Cousin Island (n = 1321 partnerships/15 years for insect abundance and territory quality) as predicted by climate window analyses. Table S2. Associations between the probability of divorce in the Seychelles warbler on Cousin Island with the total rainfall from February to August, the length of the partnership, the number of offspring, the relatedness of the breeding pair, the number of helpers, male age, female age, and population density. Data from 1997 were removed from this analysis and the non‐significant quadratic term of rainfall is included. Table S3. Associations between the probability of divorce in the Seychelles warbler on Cousin Island with the total rainfall from February to August, the length of the partnership, the number of offspring, the relatedness of the breeding pair, the number of helpers, male age, female age, and population density. Data from 1997 were removed from this analysis and the non‐significant quadratic term is excluded. Table S4. Associations between the probability of divorce in the Seychelles warbler on Cousin Island with rainfall, the length of the partnership, breeding attempted, the relatedness of the breeding pair, the number of helpers, male age, female age, and population density. Table S5. Associations between the probability of divorce in the Seychelles warbler on Cousin Island with rainfall, the length of the partnership, clutch produced, the relatedness of the breeding pair, the number of helpers, male age, female age, and population density. Table S6. Associations between the probability of divorce in the Seychelles warbler on Cousin Island with rainfall, the length of the partnership, fledgling produced, the relatedness of the breeding pair, the number of helpers, male age, female age, and population density. Table S7. Associations between the probability of divorce in the Seychelles warbler on Cousin Island wi [file JANE-94-85-s001.docx]

Supplementary materials for the manuscript titled “Rainfall is associated with divorce in the socially monogamous Seychelles warbler”.

Journal of Animal Ecology, 2024

**Materials & Methods**

**Pairwise relatedness**

Seychelles warbler DNA was extracted from brachial venipuncture blood samples using a Qiagen DNeasy Blood and Tissue Kit (2013 onwards) or modified ammonium acetate protocol (before 2013). DNA samples were used to determine sex using 1 to 3 markers and genotyping using a panel of 30 microsatellite markers (Richardson *et al.,* 2004; Raj Pant *et al.,* 2020; Sparks *et al.,* 2022). Parentage was assigned using MasterBayes 2.52 (Hadfield *et al.,* 2006), which was used to build a genetic pedigree (Sparks *et al.,* 2022). We calculated pairwise relatedness between partners using the Queller and Goodnight estimation using the R-package *related* 0.8 (Queller & Goodnight, 1989; Pew *et al.,* 2015). This estimation of pairwise relatedness also reflects pedigree relatedness in the Seychelles warbler (Brouwer *et al.,* 2007), and heterozygosity across the microsatellite panel reflects genome-wide heterozygosity (van de Crommenacker *et al.*, 2011).

**Territory quality**

Territory quality in the main breeding seasons was measured using an index of insect availability, territory size, and foliage cover (Komdeur, 1992; van de Crommenacker *et al.,* 2011). This was done using the equation *A* × ∑(*Cx × lx*), where *A* is territory size in ha, *Cx* is the percentage of foliage cover for tree species *x*, and *lx* is the per unit leaf area mean monthly insect density for tree species *x* in dm^2^*.* Insect abundance was estimated by counting the number of insects on the underside of 50 leaves for ten dominant tree species, once a month at 14 different island locations. Estimates of insect counts for all territories were estimated based on their proximity to one of these locations. Foliage cover was estimated by scoring the presence or absence of ten dominant tree species at various heights during the middle of the breeding season (typically July). This was done at 20 different points in all territories and each tree species’ total number of presence scores was its estimated foliage cover. In 2002, no territory quality data was collected resulting in 15 years of food abundance data.

**Climate window analysis**

We used *climwin* 1.2.3 (Bailey & van de Pol, 2016) to determine which temporal windows of rainfall best predicted divorce, measurements of reproductive success, and measurements of territory quality.

The *slidingwin* function determined the months of rainfall best predicting the variation in response variables using a sliding window technique. July is the peak of breeding, which is when the most eggs are laid, and warblers can then provide up to three months of post-fledgling care. As a result, we used the end of the main breeding season (28th of September) as the *slidingwin* reference date. Breeding statuses, which define our characterization of divorce, are finalized at the end of the breeding season, and our study is interested in investigating what happened in between the moment we know a partnership was last together and no longer together. As a result, we tested for all possible temporal windows (all combinations of months) from 12 months leading up to the end of the main breeding season (28th of September). Thus, from one end of the main breeding season to the end of the next.

Importantly, *climwin* is designed to avoid issues regarding multiple comparisons through a randomization scheme that ensures temporal windows are not found due to chance. Thus, after finding a temporal window, we tested whether the result was found due to chance (which was never the case) using the function *randwin*. We performed the *randwin* randomization procedure 1000 times and confirmed that observing such a negative value for the ΔAICc of the best model was statistically significant (*p*ΔAICc < 0.001).

For all response variables, both the linear and quadratic functions of rainfall were tested. AIC values of the models created were used to determine whether the linear or quadratic relationship best fit the data. A better fit for the more complicated model (quadratic) was defined as a ΔAIC > 7 (Burnham *et al.,* 2011).

**Supplementary References**

Bailey, L. D., & van de Pol, M. (2016). climwin: an R toolbox for climate window analysis. *PLOS ONE*, *11*(12), e0167980. https://doi.org/10.1371/journal.pone.0167980

Brouwer, L., Richardson, D. S., Eikenaar, C., & Komdeur, J. (2006). The role of group size and environmental factors on survival in a cooperatively breeding tropical passerine. *Journal of Animal Ecology*, *75*(6), 1321–1329. https://doi.org/10.1111/j.1365-2656.2006.01155.x

Burnham, K. P., Anderson, D. R., & Huyvaert, K. P. (2011). AIC model selection and multimodel inference in behavioral ecology: some background, observations, and comparisons. *Behavioral Ecology and Sociobiology, 65*, 23-35. https://doi.org/10.1007/s00265-010-1029-6

Hadfield, J. D., Richardson, D. S., & Burke, T. (2006). Towards unbiased parentage assignment: combining genetic, behavioural and spatial data in a Bayesian framework. *Molecular Ecology, 15*(12), 3715–3730. https://doi.org/10.1111/j.1365-294X.2006.03050.x

Komdeur, J. (1992). Importance of habitat saturation and territory quality for evolution of cooperative breeding in the Seychelles warbler. *Nature*, *358*(6386). https://doi.org/10.1038/358493a0

Pew, J., Muir, P. H., Wang, J., & Frasier, T. R. (2015). related: an R package for analysing pairwise relatedness from codominant molecular markers. *Molecular ecology resources, 15*(3), 557-561. https://doi.org/10.1111/1755-0998.12323

Raj Pant, S., Hammers, M., Komdeur, J., Burke, T., Dugdale, H. L., & Richardson, D. S. (2020). Age-dependent changes in infidelity in Seychelles warblers. *Molecular Ecology*, *29*(19), 3731–3746. https://doi.org/10.1111/mec.15563

Richardson, D. S., Komdeur, J., & Burke, T. (2004). Inbreeding in the Seychelles warbler: environment-dependent maternal effects. *Evolution; International Journal of Organic Evolution*, *58*(9), 2037–2048. https://doi.org/10.1111/j.0014-3820.2004.tb00488.x

Sparks, A. M., Spurgin, L. G., van der Velde, M., Fairfield, E. A., Komdeur, J., Burke, T., Richardson, D. S., & Dugdale, H. L. (2022). Telomere heritability and parental age at conception effects in a wild avian population. *Molecular Ecology*, *31*(23), 6324–6338. https://doi.org/10.1111/mec.15804

van de Crommenacker, J., Komdeur, J., Burke, T., & Richardson, D. S. (2011). Spatio-temporal variation in territory quality and oxidative status: a natural experiment in the Seychelles warbler (*Acrocephalus sechellensis*). *Journal of Animal Ecology*, *80*(3), 668–680. https://doi.org/10.1111/j.1365-2656.2010.01792.x

Queller, D. C., & Goodnight, K. F. (1989). Estimating relatedness using genetic markers. *Evolution, 43*(2), 258-275. https://doi.org/10.1111/j.1558-5646.1989.tb04226.x

**Results**

**Table S1.** Temporal windows of rainfall that best predict seven response variables in the Seychelles warbler on Cousin Island (*n* = 1321 partnerships/15 years for insect abundance and territory quality) as predicted by climate window analyses. The 28^th^ of September (the end of the breeding season) was set as the reference date for climate window analyses and window open/close refers to the number of months relative to this date (2 = July, 1 = August, etc…). Also presented are the function type (quadratic/linear) that best fits the model and the ΔAICc (the difference between the AICc of the model and the null model) of the model that best predicted the response variable.

| **Response variable** | **Climate variable** | | **Function type** | **ΔAICc** | **Window Open** | **Window Close** |
| --- | --- | --- | --- | --- | --- | --- |
| Insect abundance | | Rainfall | Quadratic | -394.80 | 2 | 1 |
| Territory quality | |  |  | -332.95 | 3 | 1 |
| Number of genetic fledglings surviving 3 months | |  |  | -62.94 | 5 | 0 |
| Probability of producing a fledgling | |  |  | -39.97 | 2 | 2 |
| Probability of producing a clutch | |  |  | -81.78 | 7 | 0 |
| Probability of attempting to breed | |  | Linear | -104.89 | 8 | 1 |
| Probability of divorce | |  | Quadratic | -16.89 | 7 | 1 |


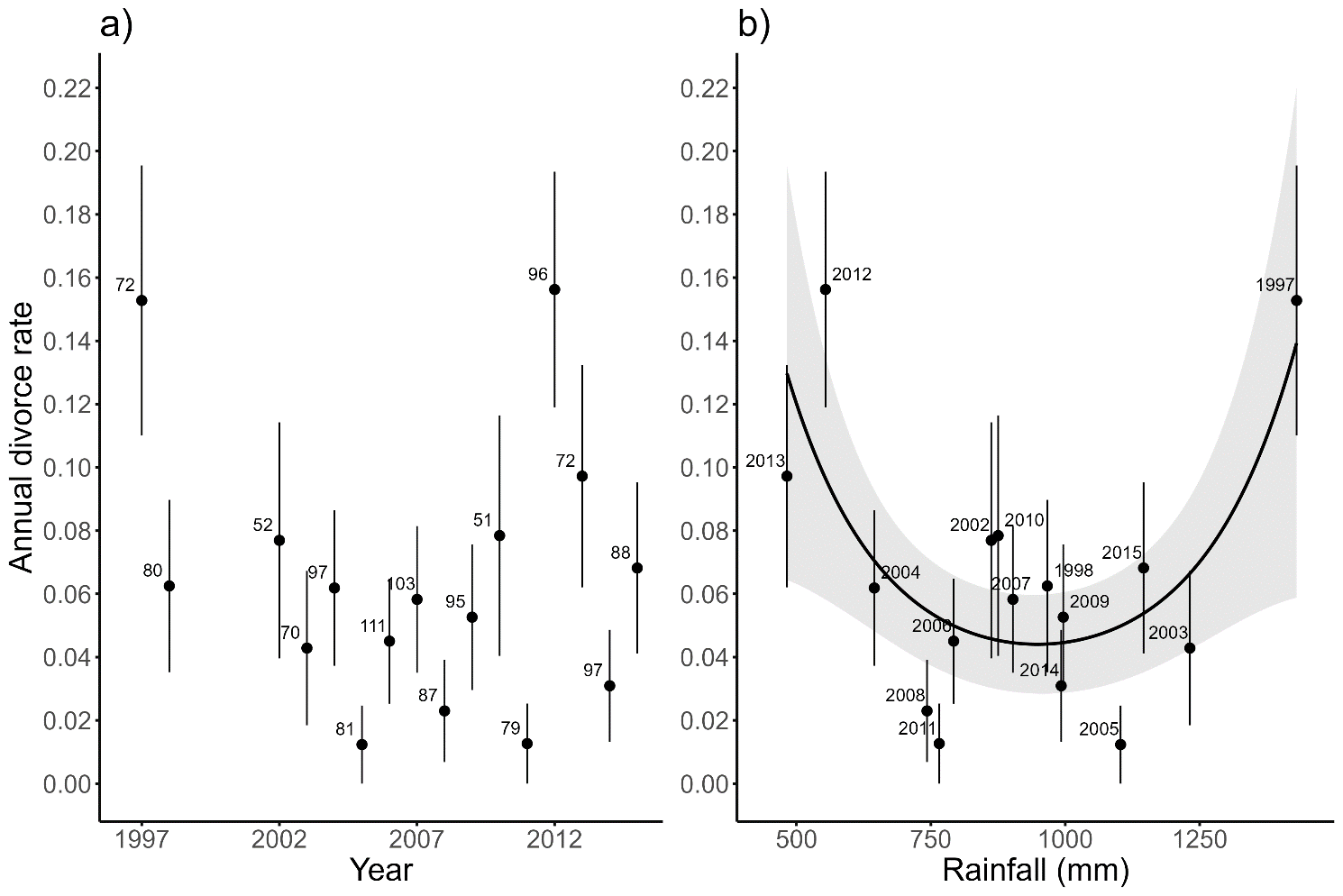


**Figure S1.** a) Variability in the annual divorce rate of the Seychelles warbler on Cousin Island (*n =* 1321 partnerships) from 1997 to 2015. The years 1999, 2000, and 2001 were not included due to limited fieldwork during those years. b) The effect of rainfall on the annual divorce rate as predicted by a quasi-binomial generalized linear model. The solid line represents the predicted divorce rate, and the grey shading indicates the 95% confidence intervals. Dots represent the mean observed annual divorce rate ± SE, and labels indicate the total number of partnerships in a given year (a) or the sample year (b).

**Table S2.** Associations between the probability of divorce in the Seychelles warbler on Cousin Island with the total rainfall from February to August, the length of the partnership, the number of offspring, the relatedness of the breeding pair, the number of helpers, male age, female age, and population density. A total of *n* = 1252 partnerships were analyzed using a binomial generalized linear mixed model. Significant *p-*values are in bold. Data from 1997 were removed from this analysis and the non-significant quadratic term of rainfall is included.

| **Independent variables** | **Estimate** | **Standard error** | **95% Confidence interval** | ***p*-value** |
| --- | --- | --- | --- | --- |
| Intercept | -4.008 | 0.696 | -5.373 to -2.644 | **<0.001** |
| Rainfall | -0.242 | 0.175 | -0.585 to 0.101 | 0.167 |
| Rainfall^2^ | 0.273 | 0.170 | -0.059 to 0.606 | 0.107 |
| Partnership length | -0.324 | 0.252 | -0.818 to 0.170 | 0.199 |
| Number of offspring | -0.102 | 0.155 | -0.406 to 0.203 | 0.513 |
| Pairwise relatedness | 0.141 | 0.161 | -0.174 to 0.456 | 0.381 |
| Number of helpers | -0.316 | 0.194 | -0.696 to 0.064 | 0.103 |
| Male age | -0.014 | 0.198 | -0.401 to 0.374 | 0.945 |
| Female age | 0.186 | 0.181 | -0.168 to 0.540 | 0.304 |
| Population density | 0.037 | 0.142 | -0.241 to 0.316 | 0.794 |
| **Random effects** | **Variance** | **Levels** |  |  |
| Male ID | 1.030 | 392 |  |  |
| Female ID | 1.168 | 372 |  |  |
| Field period ID | 0.000 | 15 |  |  |
| Territory ID | 0.182 | 156 |  |  |

**Table S3.** Associations between the probability of divorce in the Seychelles warbler on Cousin Island with the total rainfall from February to August, the length of the partnership, the number of offspring, the relatedness of the breeding pair, the number of helpers, male age, female age, and population density. A total of *n* = 1252 partnerships were analyzed using a binomial generalized linear mixed model. Significant *p-*values are in bold. Data from 1997 were removed from this analysis and the non-significant quadratic term of rainfall is excluded.

| **Independent variables** | **Estimate** | **Standard error** | **95% Confidence interval** | ***p*-value** | | |
| --- | --- | --- | --- | --- | --- | --- |
| Intercept | -3.485 | 0.818 | -5.087 to -1.882 | | **<0.001** | |
| Rainfall | -0.367 | 0.175 | -0.709 to -0.025 | | **0.036** | |
| Partnership length | -0.400 | 0.247 | -0.884 to 0.083 | | 0.105 | |
| Number of offspring | -0.098 | 0.152 | -0.396 to 0.201 | | 0.521 | |
| Pairwise relatedness | 0.114 | 0.148 | -0.177 to 0.404 | | 0.443 | |
| Number of helpers | -0.291 | 0.190 | -0.664 to 0.083 | | 0.127 | |
| Male age | -0.002 | 0.181 | -0.358 to 0.353 | | 0.989 | |
| Female age | 0.185 | 0.170 | -0.148 to 0.518 | | 0.277 | |
| Population density | 0.098 | 0.144 | -0.184 to 0.380 | | 0.497 | |
| **Random effects** | **Variance** | **Levels** |  | |  | |
| Male ID | 0.370 | 392 |  | |  | |
| Female ID | 0.550 | 372 |  | |  | |
| Field period ID | 0.052 | 15 |  | |  | |
| Territory ID | 0.310 | 156 |  | |  | |
|  |  |  |  |  | |  |


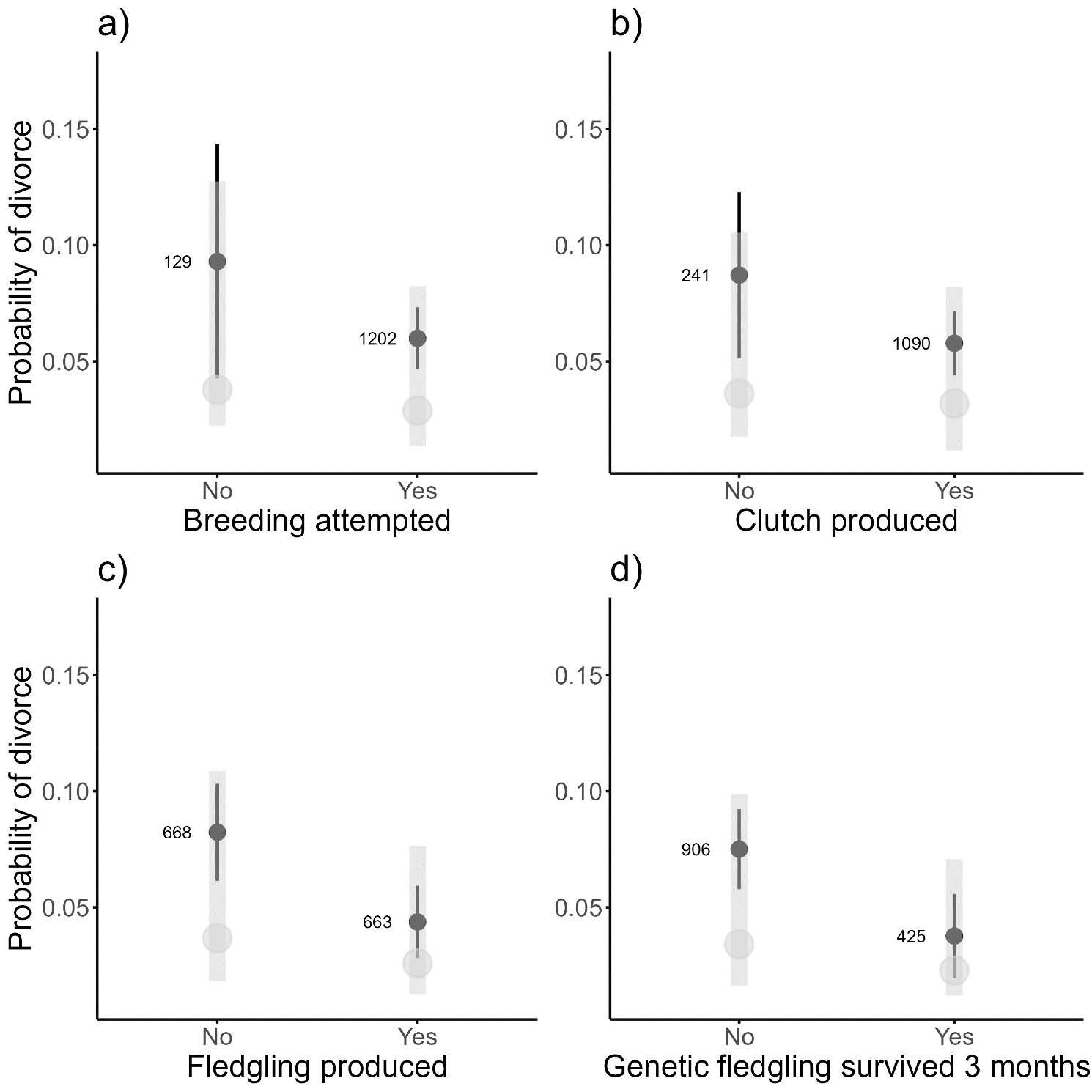


**Figure S2.** The probability of divorce for Seychelles warbler partnerships (*n =* 1321) that did or did not: a) attempt to breed; b) produce a clutch; c) produce a fledgling; d) produce a fledgling genetically related to the dominant female that survived till at the least three months old. The grey dots and shaded area represent the probability of divorce ± 95% confidence intervals as predicted by binomial generalized linear mixed model. The black dots indicate the mean observed divorce rate ± 95% confidence intervals, and labels indicate the number of partnerships.


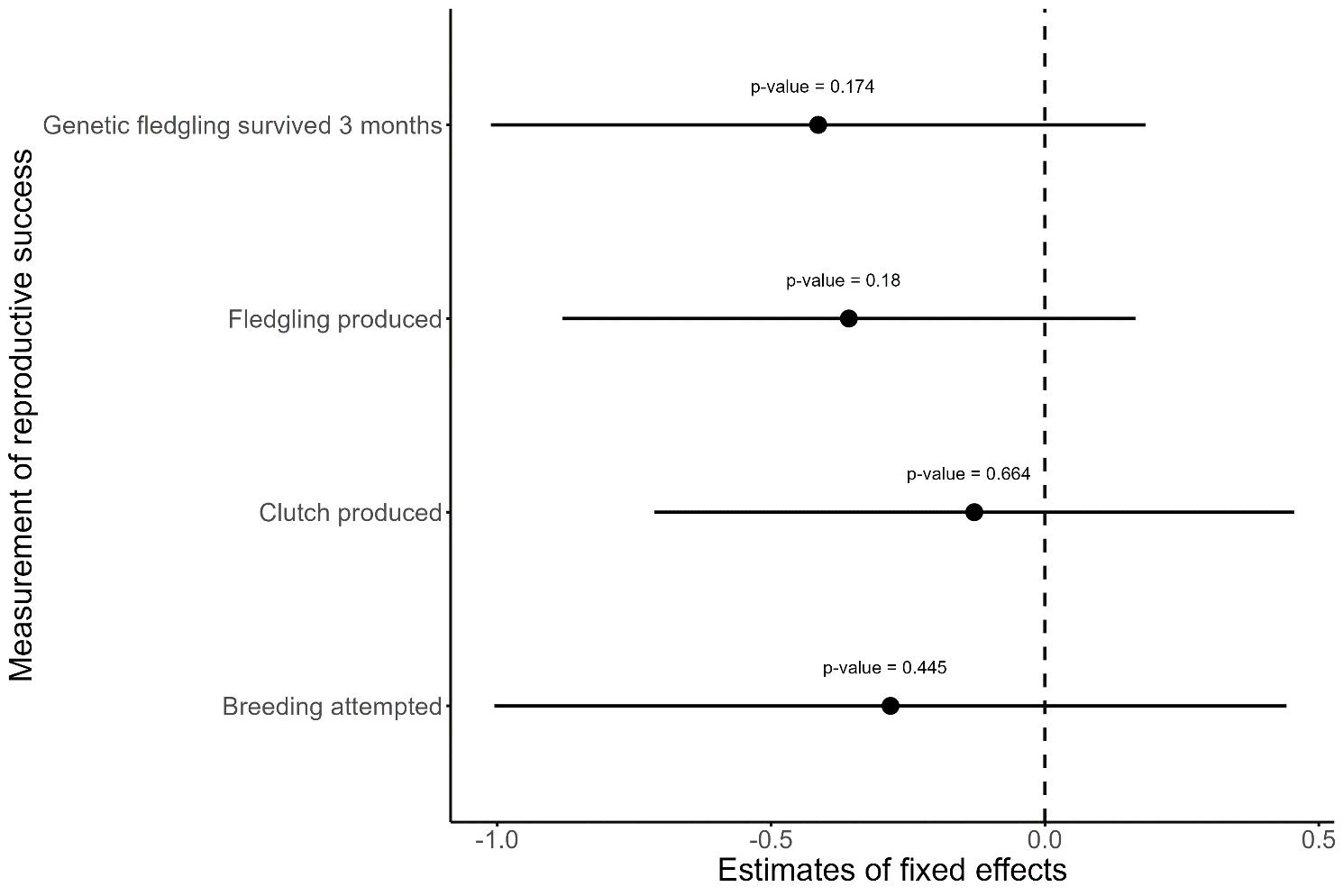


**Figure S3**. The coefficient estimates (dots) and 95% confidence intervals (bars) of four measures of reproductive success on the probability of divorce in the Seychelles warbler (*n =* 1321 partnerships) as predicted by binomial generalized linear mixed model. Each reproductive measure was independently included in the model. *p-*values are indicated on the figure.

The following tables (Tables S4 to S7) compare the effects of reproduction at four different stages - breeding attempted (Y/N), clutch produced (Y/N), fledgling produced (Y/N), and genetic fledglings (Y/N) - on divorce by including them one at a time in the partnership-level probability of divorce model. The reproductive measures are italicized in the table legends and table contents for ease of comparison between the four different model summary tables.

**Table S4.** Associations between the probability of divorce in the Seychelles warbler on Cousin Island with rainfall, the length of the partnership, *breeding attempted*, the relatedness of the breeding pair, the number of helpers, male age, female age, and population density. *n* = 1321 partnerships were analyzed using a binomial generalized linear mixed model. Significant *p*-values are in bold.

| **Independent variables** | **Estimate** | **Standard error** | **95% Confidence interval** | ***p*-value** |
| --- | --- | --- | --- | --- |
| Intercept | -3.236 | 0.469 | -4.154 to -2.317 | **<0.001** |
| Rainfall | -0.131 | 0.115 | -0.357 to 0.095 | 0.257 |
| Rainfall^2^ | 0.325 | 0.088 | 0.153 to 0.498 | **<0.001** |
| Partnership length | -0.508 | 0.217 | -0.933 to -0.084 | **0.019** |
| *Breeding attempted* | -0.282 | 0.369 | -1.006 to 0.442 | 0.445 |
| Pairwise relatedness | 0.150 | 0.128 | -0.100 to 0.401 | 0.240 |
| Number of helpers | -0.199 | 0.157 | -0.507 to 0.109 | 0.205 |
| Male age | 0.076 | 0.158 | -0.234 to 0.385 | 0.631 |
| Female age | 0.206 | 0.144 | -0.076 to 0.488 | 0.152 |
| Population density | 0.027 | 0.137 | -0.241 to 0.294 | 0.846 |
| **Random effects** | **Variance** | **Levels** |  |  |
| Male ID | <0.001 | 416 |  |  |
| Female ID | 0.272 | 392 |  |  |
| Field period ID | 0.022 | 16 |  |  |
| Territory ID | 0.353 | 158 |  |  |

**Table S5.** Associations between the probability of divorce in the Seychelles warbler on Cousin Island with rainfall, the length of the partnership, *clutch produced*, the relatedness of the breeding pair, the number of helpers, male age, female age, and population density. *n* = 1321 partnerships were analyzed using a binomial generalized linear mixed model. Significant *p*-values are in bold.

| **Independent variables** | **Estimate** | **Standard error** | **95% Confidence interval** | ***p*-value** |
| --- | --- | --- | --- | --- |
| Intercept | -3.284 | 0.351 | -3.972 to -2.596 | **<0.001** |
| Rainfall | -0.140 | 0.114 | -0.363 to 0.083 | 0.217 |
| Rainfall^2^ | 0.322 | 0.087 | 0.152 to 0.492 | **<0.001** |
| Partnership length | -0.524 | 0.208 | -0.932 to -0.115 | **0.012** |
| *Clutch produced* | -0.129 | 0.298 | -0.713 to 0.454 | 0.664 |
| Pairwise relatedness | 0.145 | 0.124 | -0.097 to 0.388 | 0.240 |
| Number of helpers | -0.199 | 0.156 | -0.505 to 0.107 | 0.203 |
| Male age | 0.080 | 0.154 | -0.222 to 0.381 | 0.605 |
| Female age | 0.203 | 0.139 | -0.068 to 0.475 | 0.143 |
| Population density | 0.026 | 0.136 | -0.241 to 0.293 | 0.848 |
| **Random effects** | **Variance** | **Levels** |  |  |
| Male ID | <0.001 | 416 |  |  |
| Female ID | <0.001 | 392 |  |  |
| Field period ID | 0.026 | 16 |  |  |
| Territory ID | 0.365 | 158 |  |  |

**Table S6.** Associations between the probability of divorce in the Seychelles warbler on Cousin Island with rainfall, the length of the partnership, *fledgling produced*, the relatedness of the breeding pair, the number of helpers, male age, female age, and population density. *n* = 1321 partnerships were analyzed using a binomial generalized linear mixed model. Significant *p*-values are in bold.

| **Independent variables** | **Estimate** | **Standard error** | **95% Confidence interval** | ***p*-value** |
| --- | --- | --- | --- | --- |
| Intercept | -3.267 | 0.363 | -3.978 to -2.556 | **<0.001** |
| Rainfall | -0.129 | 0.113 | -0.351 to 0.092 | 0.252 |
| Rainfall^2^ | 0.311 | 0.089 | 0.138 to 0.485 | **<0.001** |
| Partnership length | -0.490 | 0.215 | -0.911 to -0.069 | **0.023** |
| *Fledgling produced* | -0.358 | 0.267 | -0.882 to 0.165 | 0.180 |
| Pairwise relatedness | 0.141 | 0.127 | -0.108 to 0.390 | 0.267 |
| Number of helpers | -0.152 | 0.161 | -0.468 to 0.164 | 0.345 |
| Male age | 0.074 | 0.157 | -0.234 to 0.381 | 0.638 |
| Female age | 0.198 | 0.142 | -0.080 to 0.475 | 0.163 |
| Population density | 0.023 | 0.137 | -0.245 to 0.290 | 0.869 |
| **Random effects** | **Variance** | **Levels** |  |  |
| Male ID | 0.000 | 416 |  |  |
| Female ID | 0.187 | 392 |  |  |
| Field period ID | 0.026 | 16 |  |  |
| Territory ID | 0.334 | 158 |  |  |

**Table S7.** Associations between the probability of divorce in the Seychelles warbler on Cousin Island with rainfall, the length of the partnership, *genetic fledgling produced*, the relatedness of the breeding pair, the number of helpers, male age, female age, and population density. *n* = 1321 partnerships were analyzed using a binomial generalized linear mixed model. Significant *p*-values are in bold.

| **Independent variables** | **Estimate** | **Standard error** | **95% Confidence interval** | ***p*-value** |
| --- | --- | --- | --- | --- |
| Intercept | -3.343 | 0.341 | -4.012 to -2.674 | **<0.001** |
| Rainfall | -0.136 | 0.110 | -0.351 to 0.079 | 0.214 |
| Rainfall^2^ | 0.312 | 0.087 | 0.142 to 0.481 | **<0.001** |
| Partnership length | -0.486 | 0.216 | -0.908 to -0.063 | **0.024** |
| *Genetic fledgling produced* | -0.414 | 0.305 | -1.011 to 0.182 | 0.174 |
| Pairwise relatedness | 0.136 | 0.128 | -0.114 to 0.387 | 0.286 |
| Number of helpers | -0.196 | 0.156 | -0.502 to 0.109 | 0.208 |
| Male age | 0.075 | 0.158 | -0.234 to 0.384 | 0.634 |
| Female age | 0.190 | 0.143 | -0.090 to 0.471 | 0.184 |
| Population density | 0.019 | 0.134 | -0.244 to 0.281 | 0.890 |
| **Random effects** | **Variance** | **Levels** |  |  |
| Male ID | <0.001 | 416 |  |  |
| Female ID | 0.253 | 392 |  |  |
| Field period ID | 0.015 | 16 |  |  |
| Territory ID | 0.349 | 158 |  |  |

**Interaction between rainfall and partnership length**

We found a significant interaction between partnership length and rainfall (Table S8), where the model predicted that heavy rainfall increased the probability of divorce in shorter-lasting but not longer-lasting partnerships (Figure S4). However, as the sample sizes of longer-lasting partnerships were small (Table S9), this relationship was strongly influenced by outliers. Outliers were defined as categories with less than 50 partnerships (low estimate) or less than 100 partnerships (high estimate). In both cases, removing outliers from the analysis removed the significant interaction (Table S11; Table S12). Also, running the model with breeding experience, defined as if a partnership has been together for at least one breeding season (Y/N), instead of partnership length removed the significant interaction (Table S13). As a result, the interaction was not included in the final model.

**Table S8.** Associations between the probability of divorce in the Seychelles warbler on Cousin Island with the total rainfall from February to August, partnership length, the number of offspring, the relatedness of the breeding pair, the number of helpers, male age, female age, and population density. *n* = 1321 partnerships were analyzed using a binomial GLMM. Significant *p-*values are in bold.

| **Independent variables** | **Estimate** | **Standard error** | **95% Confidence interval** | ***p*-value** |
| --- | --- | --- | --- | --- |
| Intercept | -3.848 | 0.541 | -4.909 to -2.787 | **<0.001** |
| Rainfall | -0.347 | 0.165 | -0.671 to -0.023 | **0.036** |
| Rainfall^2^ | 0.236 | 0.118 | 0.004 to 0.467 | **0.046** |
| Partnership length | -0.624 | 0.301 | -1.215 to -0.034 | **0.038** |
| Number of offspring | -0.088 | 0.148 | -0.377 to 0.202 | 0.553 |
| Pairwise relatedness | 0.162 | 0.140 | -0.112 to 0.436 | 0.247 |
| Number of helpers | -0.196 | 0.164 | -0.519 to 0.126 | 0.232 |
| Male age | -0.073 | 0.201 | -0.466 to 0.321 | 0.717 |
| Male age^2^ | 0.191 | 0.113 | -0.030 to 0.412 | 0.090 |
| Female age | 0.274 | 0.185 | -0.087 to 0.636 | 0.137 |
| Female age^2^ | -0.122 | 0.119 | -0.356 to 0.112 | 0.306 |
| Population density | 0.039 | 0.134 | -0.224 to 0.301 | 0.774 |
| Rainfall * Partnership length | -0.486 | 0.217 | -0.912 to -0.060 | **0.025** |
| Rainfall^2^ * Partnership length | -0.063 | 0.148 | -0.354 to 0.227 | 0.670 |
| **Random effects** | **Variance** | **Levels** |  |  |
| Male ID | 0.432 | 416 |  |  |
| Female ID | 0.576 | 392 |  |  |
| Field period ID | <0.001 | 16 |  |  |
| Territory ID | 0.309 | 158 |  |  |


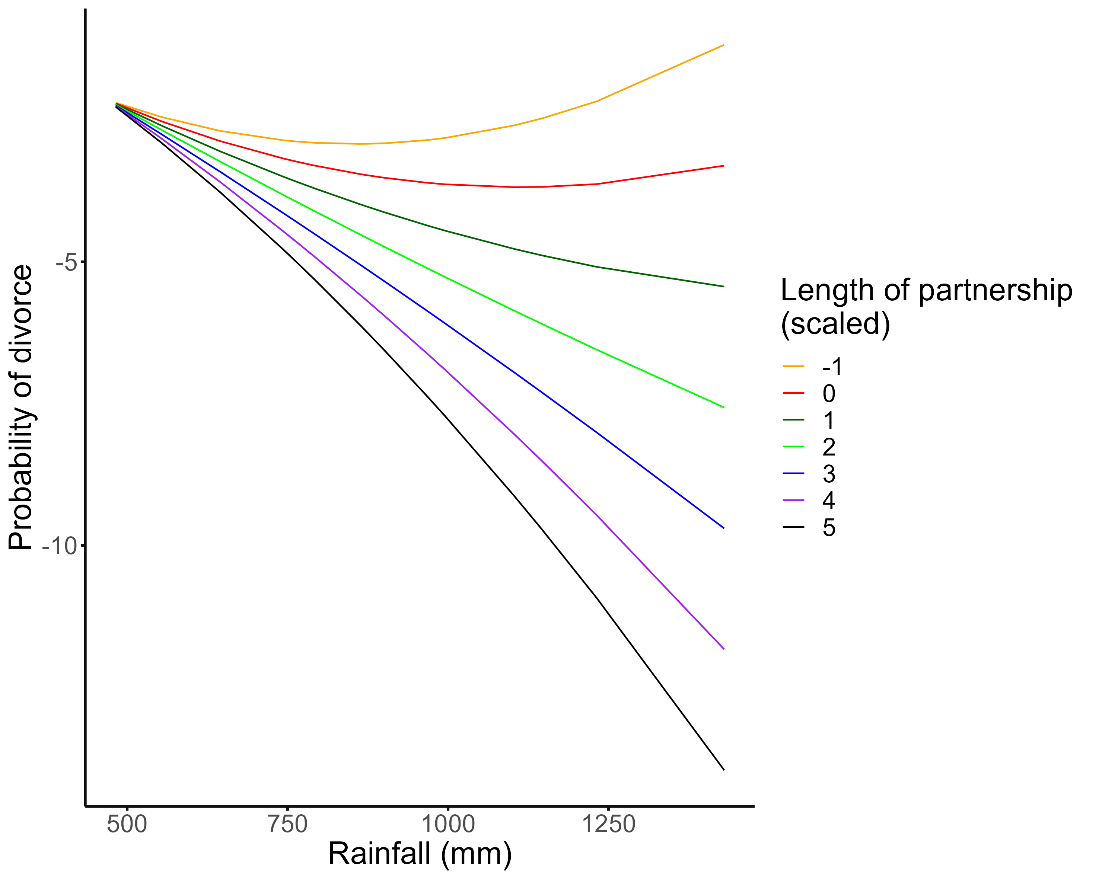


**Figure S4.** The effect of total rainfall from February to August on the probability of divorce for Seychelles warbler partnerships (*n* = 1321) that have been together for different lengths of time on Cousin Island as predicted by a binomial generalized linear mixed model.

**Table S9.** The number of available samples of Seychelles warbler partnerships that have been together for different lengths of time on Cousin Island.

| Partnership length (scaled) | Number of partnerships |
| --- | --- |
| -1 | 534 |
| 0 | 496 |
| 1 | 183 |
| 2 | 73 |
| 3 | 30 |
| 4 | 10 |
| 5 | 5 |

**Table S10.** Associations between the probability of divorce in the Seychelles warbler on Cousin Island with rainfall, the length of the partnership, the relatedness of the breeding pair, the number of helpers, male age, female age, population density, male extra-pair-paternity (EPP; infidelity), female EPP, and co-breeder presence (Y/N). *n* = 1321 partnerships were analyzed using a binomial generalized linear mixed model. Significant *p-*values are in bold.

| **Independent variables** | **Estimate** | **Standard error** | **95% Confidence interval** | ***p*-value** |
| --- | --- | --- | --- | --- |
| Intercept | -3.352 | 0.359 | -4.056 to -2.649 | **<0.001** |
| Rainfall | -0.157 | 0.113 | -0.377 to 0.064 | 0.164 |
| Rainfall^2^ | 0.324 | 0.088 | 0.152 to 0.496 | **<0.001** |
| Partnership length | -0.490 | 0.216 | -0.913 to -0.067 | **0.023** |
| Number of offspring | 0.031 | 0.217 | -0.394 to 0.456 | 0.886 |
| Pairwise relatedness | 0.136 | 0.127 | -0.112 to 0.385 | 0.283 |
| Number of helpers | -0.211 | 0.159 | -0.522 to 0.101 | 0.185 |
| Male age | 0.069 | 0.159 | -0.243 to 0.380 | 0.665 |
| Female age | 0.192 | 0.142 | -0.087 to 0.472 | 0.177 |
| Population density | 0.018 | 0.136 | -0.248 to 0.285 | 0.893 |
| Male EPP | -0.016 | 0.171 | -0.350 to 0.319 | 0.927 |
| Female EPP | -0.324 | 0.286 | -0.884 to 0.237 | 0.258 |
| Co-breeder presence | 0.412 | 0.333 | -0.240 to 1.065 | 0.215 |
| **Random effects** | **Variance** | **Levels** |  |  |
| Male ID | <0.001 | 416 |  |  |
| Female ID | 0.169 | 392 |  |  |
| Field period ID | 0.021 | 16 |  |  |
| Territory ID | 0.364 | 158 |  |  |

**Table S11.** Associations between the probability of divorce in the Seychelles warbler on Cousin Island with the total rainfall from February to August, partnership length, the number of offspring, pairwise relatedness, the number of helpers, male age, female age, and population density. *n* = 1296 partnerships were analyzed using a binomial generalized linear mixed model. Significant *p-*values are in bold. Partnerships with a partnership length greater than 3 (scaled value) were removed from this analysis.

| **Independent variables** | **Estimate** | **Standard error** | **95% Confidence interval** | | ***p*-value** | |
| --- | --- | --- | --- | --- | --- | --- |
| Intercept | -3.464 | 0.379 | | -4.206 to -2.721 | **<0.001** | |
| Rainfall | -0.299 | 0.160 | | -0.613 to 0.014 | 0.061 | |
| Rainfall^2^ | 0.247 | 0.121 | | 0.010 to 0.484 | **0.041** | |
| Partnership length | -0.849 | 0.336 | | -1.506 to -0.191 | **0.011** | |
| Number of offspring | -0.079 | 0.144 | | -0.361 to 0.204 | 0.585 | |
| Pairwise relatedness | 0.148 | 0.129 | | -0.105 to 0.401 | 0.252 | |
| Number of helpers | -0.200 | 0.165 | | -0.523 to 0.123 | 0.225 | |
| Male age | -0.011 | 0.192 | | -0.387 to 0.365 | 0.955 | |
| Male age^2^ | 0.134 | 0.116 | | -0.093 to 0.361 | 0.246 | |
| Female age | 0.327 | 0.179 | | -0.024 to 0.677 | 0.068 | |
| Female age^2^ | -0.297 | 0.151 | | -0.593 to 0.000 | 0.050 | |
| Population density | 0.077 | 0.136 | | -0.189 to 0.344 | 0.569 | |
| Rainfall * Partnership length | -0.360 | 0.214 | | -0.779 to 0.059 | 0.093 | |
| Rainfall^2^ * Partnership length | -0.028 | 0.159 | | -0.340 to 0.284 | 0.860 | |
| **Random effects** | **Variance** | **Levels** | |  |  | |
| Male ID | 0.000 | 416 | |  |  | |
| Female ID | 0.302 | 392 | |  |  | |
| Field period ID | 0.013 | 16 | |  |  | |
| Territory ID | 0.291 | 158 | |  |  | |
|  |  |  | |  |  |  |

**Table S12**. Associations between the probability of divorce in the Seychelles warbler on Cousin Island with the total rainfall from February to August, partnership length, the number of offspring, pairwise relatedness, the number of helpers, male age, female age, and population density. *n* = 1245 partnerships were analyzed using a binomial generalized linear mixed model. Significant *p-*values are in bold. Partnerships with a partnership length greater than 2 (scaled value) were removed from this analyses.

| **Independent variables** | **Estimate** | **Standard error** | **95% Confidence interval** | ***p*-value** | |
| --- | --- | --- | --- | --- | --- |
| Intercept | -3.477 | 0.389 | -4.240 to -2.714 | **<0.001** | |
| Rainfall | -0.280 | 0.161 | -0.595 to 0.034 | 0.081 | |
| Rainfall^2^ | 0.240 | 0.123 | -0.001 to 0.482 | 0.051 | |
| Partnership length | -0.790 | 0.353 | -1.482 to -0.099 | **0.025** | |
| Number of offspring | -0.110 | 0.147 | -0.398 to 0.178 | 0.453 | |
| Pairwise relatedness | 0.128 | 0.130 | -0.127 to 0.383 | 0.324 | |
| Number of helpers | -0.194 | 0.165 | -0.518 to 0.130 | 0.241 | |
| Male age | -0.029 | 0.194 | -0.409 to 0.351 | 0.880 | |
| Male age^2^ | 0.152 | 0.119 | -0.082 to 0.386 | 0.204 | |
| Female age | 0.323 | 0.178 | -0.026 to 0.673 | 0.070 | |
| Female age^2^ | -0.268 | 0.153 | -0.569 to 0.033 | 0.081 | |
| Population density | 0.082 | 0.131 | -0.176 to 0.339 | 0.535 | |
| Rainfall * Partnership length | -0.337 | 0.230 | -0.789 to 0.114 | 0.143 | |
| Rainfall^2^ * Partnership length | -0.035 | 0.173 | -0.374 to 0.303 | 0.838 | |
| **Random effects** | **Variance** | **Levels** |  |  | |
| Male ID | 0.000 | 416 |  |  | |
| Female ID | 0.325 | 392 |  |  | |
| Field period ID | 0.000 | 16 |  |  | |
| Territory ID | 0.284 | 158 |  |  | |
|  |  |  |  |  |  |

**Table S13.** Associations between the probability of divorce in the Seychelles warbler on Cousin Island with the total rainfall from February to August, breeding experience, the number of offspring, the relatedness of the breeding pair, the number of helpers, male age, female age, and population density. *n* = 1321 partnerships were analyzed using a binomial generalized linear mixed model. Significant *p-*values are in bold.

| **Independent variables** | **Estimate** | **Standard error** | | **95% Confidence interval** | ***p*-value** | |
| --- | --- | --- | --- | --- | --- | --- |
| Intercept | -3.248 | | 0.664 | -4.549 to -1.946 | | **<0.001** |
| Rainfall | 0.013 | | 0.159 | -0.298 to 0.324 | | 0.936 |
| Rainfall^2^ | 0.240 | | 0.118 | 0.009 to 0.471 | | **0.042** |
| Breeding experience | -0.988 | | 0.429 | -1.829 to -0.146 | | **0.021** |
| Number of offspring | -0.077 | | 0.148 | -0.367 to 0.213 | | 0.604 |
| Pairwise relatedness | 0.160 | | 0.140 | -0.114 to 0.435 | | 0.252 |
| Number of helpers | -0.201 | | 0.163 | -0.520 to 0.119 | | 0.218 |
| Male age | -0.127 | | 0.191 | -0.501 to 0.247 | | 0.507 |
| Male age^2^ | 0.164 | | 0.115 | -0.061 to 0.389 | | 0.154 |
| Female age | 0.226 | | 0.177 | -0.120 to 0.572 | | 0.200 |
| Female age^2^ | -0.138 | | 0.121 | -0.374 to 0.099 | | 0.254 |
| Population density | 0.036 | | 0.134 | -0.227 to 0.298 | | 0.790 |
| Rainfall * Breeding experience | -0.334 | | 0.233 | -0.790 to 0.123 | | 0.152 |
| Rainfall^2^ * Breeding experience | 0.215 | | 0.171 | -0.120 to 0.550 | | 0.208 |
| **Random effects** | **Variance** | | **Levels** |  | |  |
| Male ID | 0.434 | | 416 |  | |  |
| Female ID | 0.575 | | 392 |  | |  |
| Field period ID | 0.000 | | 16 |  | |  |
| Territory ID | 0.317 | | 158 |  | |  |

**Table S14.** Associations between the probability of attempting to breed (model 1), producing a clutch (model 2), producing a fledgling (model 3), and the number of genetic fledglings surviving till at least three months old (model 4) in the Seychelles warbler on Cousin Island with rainfall, partnership length, pairwise relatedness, the number of helpers, male age, and female age. *n* = 1321 partnerships were analyzed using binomial (models 1 to 3) and poisson (model 4) generalized linear mixed models. Significant terms are in bold.

|  | **Model 1** | **Model 2** | **Model 3** | **Model 4** | |
| --- | --- | --- | --- | --- | --- |
| **Independent variables** | **Estimate**  **(95% Confidence interval)** | | | |  |
| Intercept | **3.44*****  **(2.72 to 4.16)** | **2.63*****  **(2.11 to 3.14)** | **0.79*****  **(0.41 to 1.17)** | **-0.54*****  **(-0.80 ­to -0.27)** | |
| Rainfall | **0.948****  **(0.37 to 1.53)** | **0.61****  **(0.23 to 1.00)** | **0.33****  **(0.06 to 0.59)** | **0.20***  **(0.01 to 0.41)** | |
| Rainfall^2^ | - | - | **-0.23***  **(-0.44 to -0.05)** | **-0.22****  **(-0.38 to -0.06)** | |
| Partnership length | -0.16  (-0.50 to 0.19) | 0.09  (-0.17 to 0.35) | 0.14  (-0.07 to 0.35) | 0.04  (-0.11 to 0.18) | |
| Pairwise relatedness | 0.00  (-0.24 to 0.24) | -0.03  (-0.21 to 0.14) | **-0.14***  **(-0.28 to 0.00)** | -0.08  (-0.17 to 0.01) | |
| Number of helpers | - | **1.49*****  **(0.86 to 2.13)** | **0.76*****  **(0.58 to 0.94)** | **0.09***  **(0.02 to 0.17)** | |
| Male age | **0.47***  **(0.11 to 0.83)** | 0.10  (-0.12 to 0.33) | **0.22***  **(0.01 to 0.43)** | **0.21****  **(0.06 to 0.35)** | |
| Male age^2^ | **-0.23***  **(-0.41 to -0.05)** | - | **-0.19****  **(-0.31 to -0.08)** | **-0.16*****  **(-0.25 to -0.07)** | |
| Female age | 0.26  (-0.03 to 0.55) | **0.37****  **(0.14 to 0.61)** | 0.16  (-0.04 to 0.36) | 0.03  (-0.12 to 0.17) | |
| Female age^2^ | - | **-0.30*****  **(-0.44 to -0.17)** | **-0.28*****  **(-0.39 to -0.16)** | **-0.21*****  **(-0.32 to -0.12)** | |
| **Random effects (Levels)** | **Variance** | **Variance** | **Variance** | **Variance** | |
| Male ID (416) | 0.00  0.34  1.03  0.43 | 0.00  0.00  0.51  0.35 | 0.02  0.22  0.21  0.07 | 0.00  0.00  0.12  0.00 | |
| Female ID (392) |  |  |  |  |  |
| Field period ID (16) |  |  |  |  |  |
| Territory ID (158) |  |  |  |  |  |

*: *p*-value < 0.05; **: *p*-value = 0.001: ***: *p*-value < 0.001

**Table S15.** Associations between the probability of producing a clutch in the Seychelles warbler on Cousin Island with total rainfall from February to August, the length of the partnership, the relatedness of the breeding pair, the number of helpers, male age, and female age. *n* = 1321 partnerships were analyzed using a binomial generalized linear mixed model. Significant *p-*values are in bold. The non-significant quadratic term of rainfall is included.

| **Independent variables** | **Estimate** | **Standard error** | **95% Confidence interval** | ***p*-value** |
| --- | --- | --- | --- | --- |
| Intercept | 2.899 | 0.294 | 2.322 to 3.476 | **<0.001** |
| Rainfall | 0.682 | 0.182 | 0.325 to 1.038 | **<0.001** |
| Rainfall^2^ | -0.264 | 0.136 | -0.530 to 0.002 | 0.051 |
| Partnership length | 0.084 | 0.134 | -0.178 to 0.347 | 0.528 |
| Pairwise relatedness | -0.034 | 0.088 | -0.206 to 0.138 | 0.702 |
| Number of helpers | 1.499 | 0.322 | 0.868 to 2.131 | **<0.001** |
| Male age | 0.100 | 0.114 | -0.123 to 0.324 | 0.378 |
| Female age | 0.373 | 0.121 | 0.135 to 0.611 | 0.002 |
| Female age^2^ | -0.303 | 0.069 | -0.438 to -0.167 | **<0.001** |
| **Random effects** | **Variance** | **Levels** |  |  |
| Male ID | <0.001 | 416 |  |  |
| Female ID | 0.000 | 392 |  |  |
| Field Period ID | 0.403 | 16 |  |  |
| Territory ID | 0.355 | 158 |  |  |


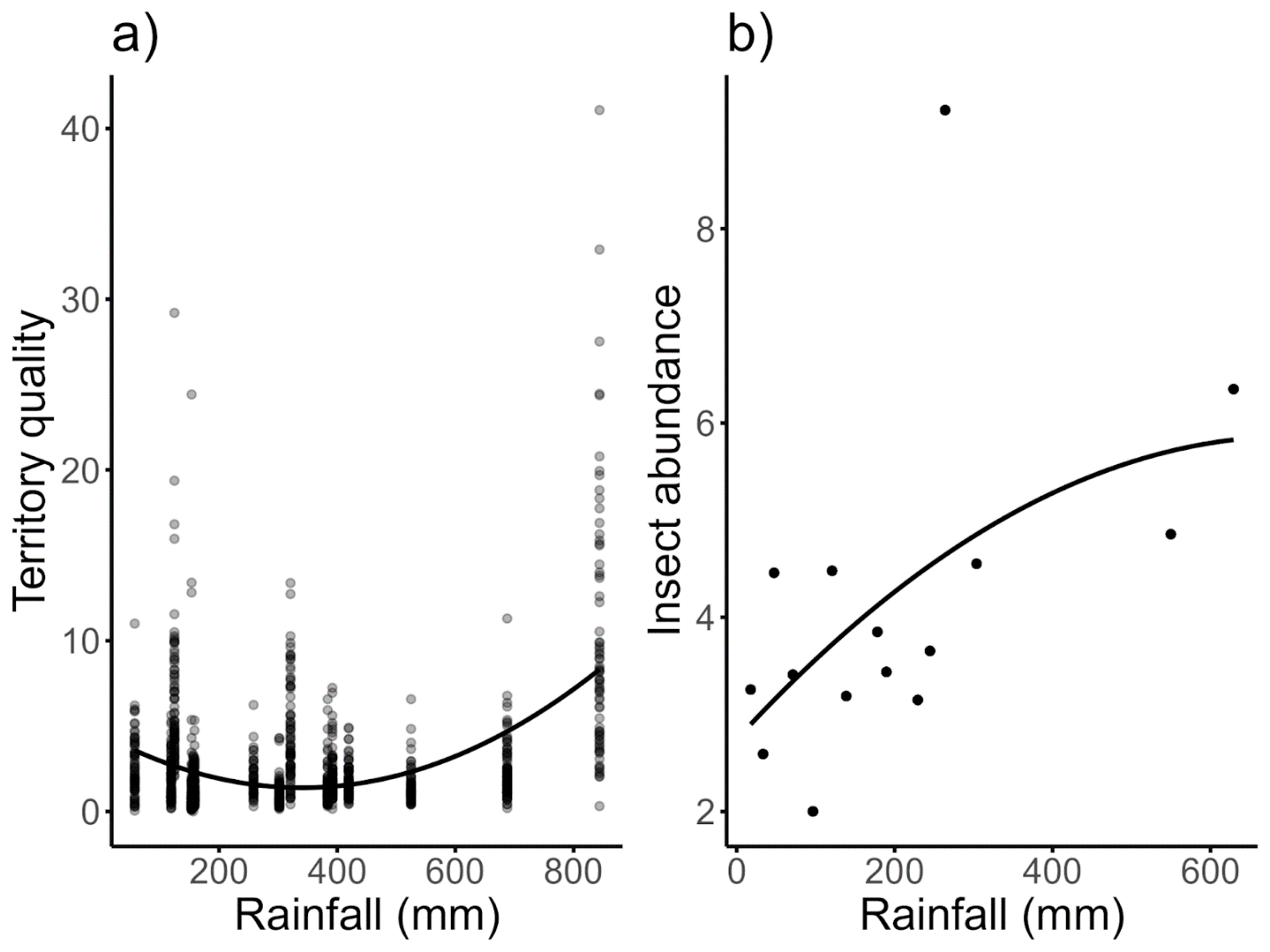
**Figure S5.** The effect of rainfall on: a) territory quality (scaled 1:10,000); b) insect abundance (the mean number of insects found per unit leaf area across all monthly surveys) on Cousin Island (*n =* 15 years), as predicted (solid line) by a *climwin* generated linear model. Territory quality was best predicted by rainfall from June to August, and insect abundance was best predicted by rainfall from July to August. Data are indicated as points where the shade of the points represents the sample size (darker represents more samples).
